# Chemical Bond‐Orchestrated Energy Depletion to Augment Lung Cancer Chemo‐Immunotherapy

**DOI:** 10.1002/advs.77135

**Published:** 2026-08-11

**Authors:** Xuejie Zhao, Yuting Liu, Xiao Huang, Liwen Zhao, Chunli Li, Yongwei Huang

**Affiliations:** ^1^ Laboratory for NanoMedical Photonics School of Basic Medical Science Henan University Kaifeng People's Republic of China

**Keywords:** chemical bond engineering, chemo‐immunotherapy, glycolysis and OXPHOS, PDHKs inhibition, perylenediimide, reactive oxygen species

## Abstract

Concurrent blockade of aerobic glycolysis and oxidative phosphorylation (OXPHOS) holds great promise in lung cancer therapy yet challenged by tumor cell metabolic plasticity. To address this, we herein grafted dichloroacetic acid into perylenediimide (PDI) skeleton via ionic or covalent bond to create PDIC‐AC and PDIC‐NAC. Studies demonstrate that ionic bond‐driven primary amine positive nitrogen remodeling and mitochondrial localization endow PDIC‐AC with significantly stronger inhibitory activity on pyruvate dehydrogenase kinases (PDHKs) than PDIC‐NAC. Notably, PDIC‐AC targets the Rieske iron‐sulfur polypeptide 1 (UQCRFS1) subunit of complex III in mitochondria, triggering electron leakage from the electron transport chain, thereby more efficiently inducing reactive oxygen species (ROS) production relative to PDIC‐NAC. Superior PDHKs inhibiting efficacy and ROS generation capacity functionalize PDIC‐AC as an efficient inhibitor to block glycolysis and OXPHOS, which not only repolarize macrophages toward anti‐tumor M1 phenotype via suppression of lactate production, but also trigger immunogenic cell death via PERK‐eIF2α‐ATF4‐CHOP axis to activate immune response, ultimately reaching effective chemo‐immunotherapy against the primary and distant tumors. Overall, this work defines the unambiguous mechanism for PDI‐triggered endogenous ROS generation, and meanwhile clarifies small‐molecule regulators' energy metabolism intervention mechanism and establishes an innovative chemical bond engineering strategy for energy‐targeted chemo‐immunotherapy.

## Graphical abstract

This study grafted dichloroacetic acid (DCA) into perylenediimide (PDI) skeleton via ionic or covalent bond to create novel metabolic agent PDIC‐AC and PDIC‐NAC, explicitly elaborated the underlying energy metabolism and therapeutic mechanism, and developed a promising strategy for energy metabolism‐targeting chemo‐immunotherapy by chemical bond engineering.

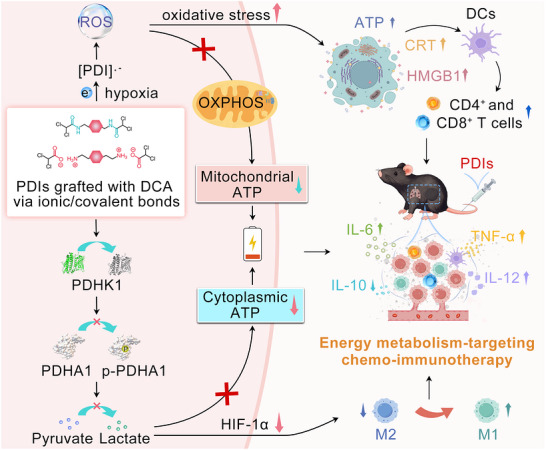

## 1 Introduction

Cancer cells require sufficient energy to meet increased demands on proliferation and division. Aerobic glycolysis, also known as the Warburg effect, is the predominant energy production pathway used by most tumors [1, 2, 3, 4]. In glycolysis, adenosine triphosphate (ATP) was efficiently generated to meet the nutrient requirements of cancer cells, and meanwhile the resultant lactate (LA) can cause severe immunosuppression to discount anti‐tumor effect [5, 6, 7]. Therefore, targeting glycolysis to cut off the energy supply has emerged as a prospective strategy in the fight against cancer [8, 9]. However, the anti‐tumor efficacy of single glycolysis inhibition remains unsatisfactory due to the presence of metabolic heterogeneity and compensatory properties, where tumor cells typically switch metabolic phenotypes between glycolysis and oxidative phosphorylation (OXPHOS) to compensate for energy supply [10, 11, 12, 13]. Thus, simultaneously intervening glycolysis as well as OXPHOS via cooperation mechanisms might be a powerful and promising strategy for boosting energy deprivation and dismantling the immunosuppressive microenvironment. In this context, versatile nanoplatforms such as metformin co‐loaded CuFe_2_O_4_ nanoparticles, Zn−carnosine metallodrug, proximities nanogenerator APAP‐P‐NO have been developed for blocking glycolysis and OXPHOS to reprogram tumor metabolism process, confirming the effectiveness and necessity of synergetic energy deprivation [14, 15, 16, 17]. However, despite that these currently available smart systems present attractive and valuable paradigms for boosting cancer therapy, they still obsessed by the single‐pathway action, complicated fabrication and uncontrollable properties [18, 19, 20]. Considering these obstacles, single small molecular prodrugs with dual‐inhibition of glycolysis and OXPHOS may present more simpler yet efficient platform to block energy supply in tumor cells.

Mechanistically, glycolysis is cytoplasmic‐based and OXPHOS is mitochondria‐based metabolic process [21, 22, 23]. The inherent spatiotemporal differences allow the design of a single small molecule drug with dual‐inhibition of glycolysis and OXPHOS still keep challenging. Fortunately, dichloroacetic acid (DCA), the only pyruvate dehydrogenase kinases (PDHKs) inhibitor that enters phase II clinical trials, has been shown to bind to the pyruvate binding site of PDHKs, thereby reversing the Warburg effect effectively [24, 25]. Also, extensive studies demonstrated that elevated reactive oxygen species (ROS) level can impair mitochondrial OXPHOS in multiple respects [26, 27]. Given this, it is proposed that the conjugation of DCA into ROS generator may efficiently achieve dual‐inhibition of glycolysis and OXPHOS to impede cellular energy production. Notably, the electron cloud density within DCA‐based inhibitor significantly impacts its binding efficacy to PDHKs [28, 29]. Specifically, differences in electronegativity can alter the spatial conformation or electrostatic complementarity of inhibitor to affect its binding site and binding energy with PDHKs [30]. In addition, electron cloud density has been shown to regulate the ROS production efficiency [31, 32]. Currently, electron‐donating/withdrawing groups or heavy atoms have been widely incorporated by covalent bonds to modulate the binding efficacy to PDHKs or ROS production ability [28, 29, 33, 34, 35]. Comparatively, ionic bond‐orchestrated energy metabolism and ROS production have received less attention. In this context, concatenating DCA into ROS generator via ionic bond might engineer an innovative and promising paradigm to reprogram energy metabolism.

Recently, phenylenediamine (PDI) derivatives have received comprehensive attention in biomedical field [36, 37, 38, 39, 40]. PDI scaffold contains multiple reaction sites that not only are favorable to introduction of DCA to directly bind PDHK protein but also to form an extensive hydrogen bond network with PDHK protein to cooperatively achieve enzyme inhibition. More importantly, PDI scaffold has been vindicated to undergo persistent radical species process to enable sustained ROS production in tumor cells [41, 42], although the unambiguous mechanism still keep unknown to date. Thus, PDI derivative can be functionalized as a conceptual candidate to reach effective chemo‐immunotherapy against tumors via dual‐inhibition of glycolysis and OXPHOS.

**SCHEME 1:**
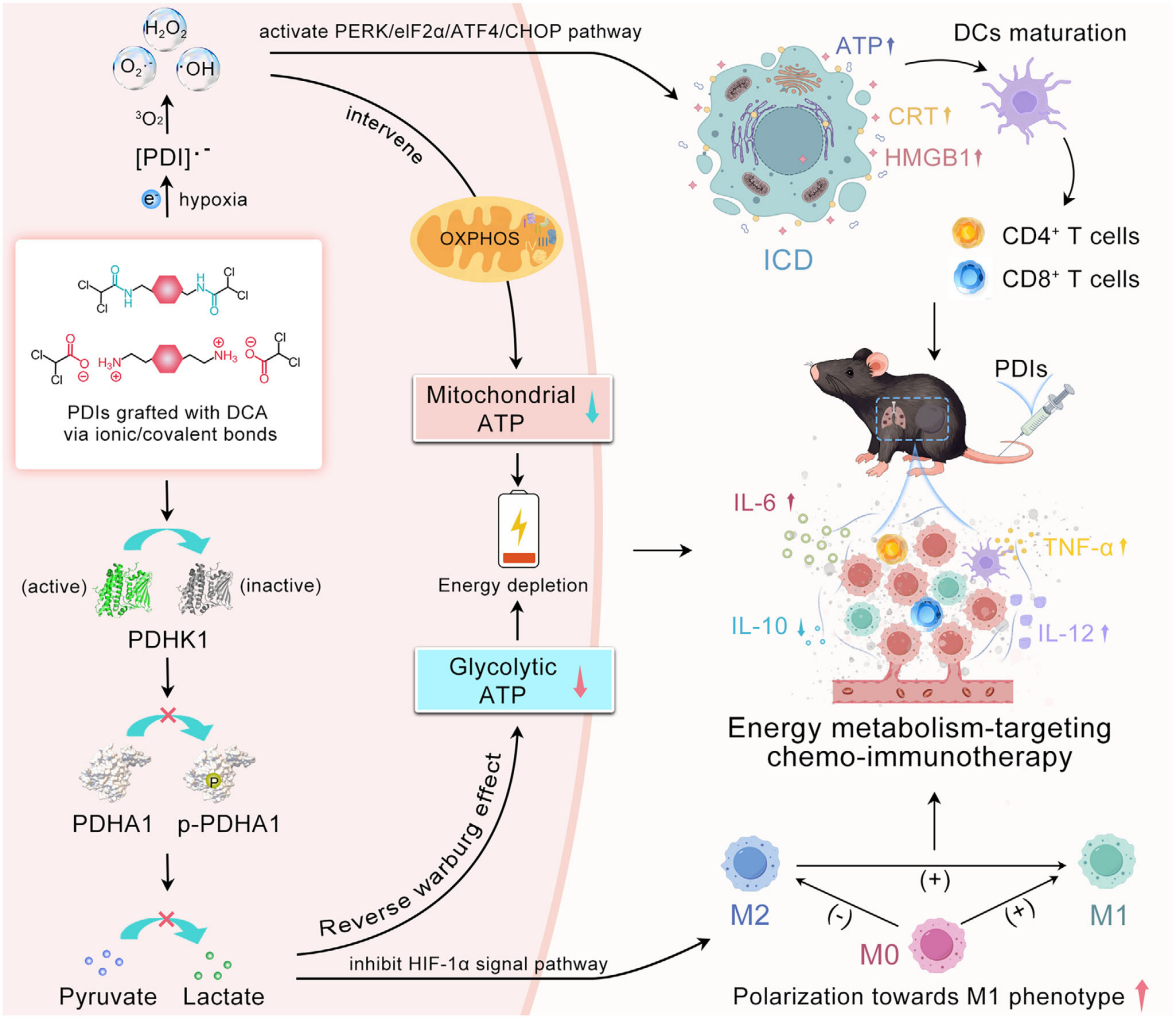
The mechanism of chemical bond engineering‐orchestrated the inhibition of glycolysis and oxidative phosphorylation to augment lung cancer chemo‐immunotherapy.

Inspired by these findings, we herein grafted DCA into terminus of primary amines by ionic or covalent bond to obtain PDIC‐AC and PDIC‐NAC, respectively. To our surprise, ionic and covalent bond‐orchestrated the reshaping of molecular scaffold and electron distribution allow PDIC‐AC and PDIC‐NAC to show remarkably differential inhibition activity to PDHKs. Specifically, PDIC‐NAC can only bind to the lipoamide domain of PDHKs via dichloroacetamide chain. By contrast, the positive nitrogen in primary amine and DCA anion allow PDIC‐AC to simultaneously bind to lipoamide and pyruvate domain of PDHKs, thereby achieving the dual inhibition effect on PDHKs. More fascinating, we demonstrated that PDIC‐AC induces electron leakage from the electron transport chain (ETC) by interfering with Rieske iron‐sulfur polypeptide 1 (UQCRFS1) subunit of complex III. PDIC‐AC captures the leaked electrons and is reduced to delocalized [PDIC‐AC]^•−^ to initiate ROS generation. In contrast, PDIC‐NAC primarily captures electrons from nicotinamide adenine dinucleotide phosphate hydrogen (NADPH)‐cytochrome P450 system in the cytoplasm to form [PDIC‐NAC]^•−^, which results in less efficient ROS production than PDIC‐AC, establishing the first unambiguous mechanism for PDI‐triggered endogenous ROS generation in hypoxic cells. Superior inhibiting efficacy on PDHKs and ROS generation capacity functionalize PDIC‐AC as an efficient inhibitor to block the glycolysis and OXPHOS in tumor cell, which not only repolarize promoting‐tumor M2 macrophages into anti‐tumor M1 phenotype by curtail LA production, but also trigger immunogenic cell death (ICD) by PERK‐eIF2α‐ATF4‐CHOP signaling pathway to activate immune response (Scheme 1), ultimately reaching effective chemo‐immunotherapy on the primary and distant lung tumors. Overall, taking PDI as a model, this work not only explicitly elaborates the energy metabolism and therapeutic mechanism of small molecular metabolic agent, but also develops an innovative and promising strategy for energy metabolism‐targeting chemo‐immunotherapy by chemical bond engineering.

## 2 Results and Discussion

### 2.1 Synthesis, Characterization, and Physical Properties of PDIC‐AC

**FIGURE 1:**
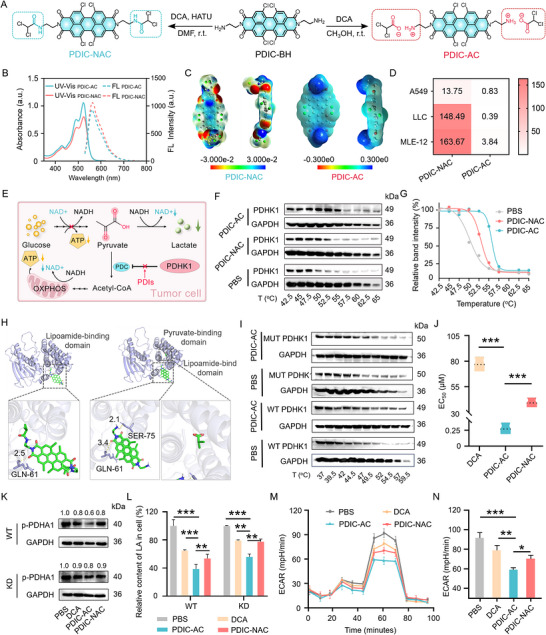
Synthesis, physical properties, and PDHK1 inhibitory activity of the compounds. (A) The molecular structure of PDIC‐BH, PDIC‐AC and PDIC‐NAC. (B) The UV–vis absorption spectrum (40 µm) and fluorescence (FL) emission spectrum (3.33 µm) of PDIC‐AC in water and PDIC‐NAC in water containing 1% DMSO. (C) Optimized electrostatic potential of PDIC‐NAC (left) and PDIC‐AC+ (right) by DFT calculation based on the B3LYP/6‐31G (d, p) (C: gray, H: white, O: red, N: blue, Cl: green). Blue and red regions indicate a relatively positive and negative charge distribution, respectively. (D) IC_50_ of PDIC‐NAC and PDIC‐AC on A549, LLC and MLE‐12 cells. (E) Schematic diagram of glycolysis and OXPHOS metabolism. (F) PDHK1 expression treated by PDIC‐AC or PDIC‐NAC in LLC cells and (G) quantitative result of the PDHK1 protein thermal stability. (H) Molecular docking of PDIC‐NAC (left) and PDIC‐AC (right) with PDHK1 kinase (PDB code: 2Q8G). (I) CETSA results of Flag tagged WT‐PDHK1 and Flag tagged MUT PDHK1 protein treated by PDIC‐AC. (J) Inhibition effect of DCA, PDIC‐AC, and PDIC‐NAC on PDHK1 enzyme activity. (K) Expression levels of p‐PDHA1 and (L) intracellular LA content in WT and KD LLC cells after treatment of DCA, PDIC‐AC and PDIC‐NAC. (M) Glycolytic function assessed by seahorse glycolysis stress test and (N) maximal glycolytic capacity in LLC cells treated with DCA (2 mm), PDIC‑AC (2 µm) and PDIC‑NAC (2 µm). Data represent mean ± SD (*n* = 3). Statistical comparisons were performed using one‐way ANOVA. *p < 0.05, **p < 0.01, ***p < 0.001.

1, 6, 7, 12‐tetrachloro‐3, 4, 9, 10‐perylenetetracarboxylic acid dianhydride (530 mg, 1.00 mmol) and *N*‐(tert‐butoxy carbonyl)‐1, 2‐diaminohexane (*N*‐Boc‐1, 2‐diaminohexane) (480 µL, 3.00 mmol, 3.0 equiv) were dissolved in isopropanol (20 mL) and reacted for 12 h at 80°C under argon atmosphere to obtain compound 1 (643 mg, 0.79 mmol, 79% yield) (Figure S1). Then, compound 1 (240 mg, 0.29 mmol) reacted with trifluoroacetic acid (6 mL, 78 mmol, 259.0 equiv) in dichloromethane (6 mL) at room temperature for 6 h to remove tert‐butoxy carbonyl groups. The resultant solid product was collected by vacuum rotary evaporation and recrystallized from diethyl ether to achieve the compound 2 (Figure S1). Subsequently, dispersing compound 2 in 5% sodium carbonate aqueous solution to remove trifluoroacetic acid, and the resultant product was washed with distilled water and dried under vacuum to obtain key intermediate product PDIC‐BH (134 mg, 0.22 mmol, 75% yield) (Figure 1A; Figure S1). PDIC‐BH (50.3 mg, 0.08 mmol) was dissolved in methanol (10 mL) and acidified by DCA (20 µL, 0.24 mmol, 3.0 equiv) to give *N, N’*‐Bis (2‐(aminoethyl) ethylene) perylene‐1, 6, 7, 12‐tetrachloro‐3, 4, 9, 10‐tetracarboxyldiimide dichloroacetate (PDIC‐AC) (43.2 mg, 0.05 mmol, 61% yield) (Figure 1A and Figure S1). *N, N’*‐Bis (2‐(dichloroacetamide) ethylene) perylene‐1, 6, 7, 12‐tetrachloro‐3, 4, 9, 10‐tetracarboxyldiimide (PDIC‐NAC) was subsequently prepared by the acylation reaction between PDIC‐BH (50 mg, 0.08 mmol) and DCA (14 µL, 0.17 mmol, 2.1 equiv) with the catalysis of 2‐(7‐azobenzotriazolyl)‐*N, N, N', N'*‐tetramethyluronium hexafluorophosphate (HATU)(92.5 mg, 0.24 mmol, 3.0 equiv) to afford the target compound (38.2 mg, 0.57 mmol) in 57% yield (Figure 1A and Figure S1). The detailed synthesis process and characterization of PDIC‐AC and PDIC‐NAC using ^1^H NMR, ^13^C NMR, and high‐resolution electrospray ionization mass spectrometry (ESI‐MS) were shown in Figures S1–S7.

We subsequently acquired absorption and fluorescence (FL) emission spectra of PDIC‐AC and PDIC‐NAC with UV–Visible (UV–Vis) spectrophotometer and FL spectrofluorometer, respectively. As shown in Figure 1B, PDIC‐NAC and PDIC‐AC exhibited characteristic absorption peaks predominantly at 432, 488, and 525 nm, which represent typical single‐molecular spectral features. Upon excitation at 525 nm, both PDIC‐NAC and PDIC‐AC exhibit a strong fluorescence emission at 570 nm (Figure 1B), providing highly favorable conditions for tracking their distribution in cells and in biological tissue. Being different from the absorption and emission spectra, computational analysis using Gaussian 09 reveals a distinct contrast in the electrostatic potential distribution. Specifically, the positive charge (blue) of PDIC‐NAC exhibits a localized and small‐scale distribution, without forming a strongly positive region that spans the molecule. In contrast, the positive charge of PDIC‐AC is concentrated in large regions at both ends of the molecule with significant enrichment along the edges of the aromatic backbone. This results in a broader and more intense positively charged area, presenting a more pronounced “positive‐charged ends” characteristic (Figure 1C and Figure S8). These differences affect the electrostatic complementarity with the binding pocket of the target protein, thereby modulating its activity.

To investigate the stability of PDIC‐AC under physiological conditions, we first determined the acid dissociation constant (pKa) of the compound, which was measured to be 7.15 (Figure S9A). Based on the Henderson‐Hasselbalch equation (pKa = pH + log([HA]/[A^−^])) [43], approximately 64% of PDIC‐AC exists in its dissociated form at physiological pH 7.4, while 36% remains undissociated. This indicates a dynamic equilibrium between the cationic PDIC‐BH^+^ and the anionic DCA^−^ in neutral solutions. To further assess the stability of ionic salts under physiological conditions, we subsequently employed dynamic light scattering (DLS) and UV‑‐vis to monitor aggregation in phosphate‐buffered saline (PBS) containing 10% fetal bovine serum (FBS) to simulate cell media at pH 7.4 (PBS+10%FBS). As suggested in Figure S9B, comparation with the single‐molecule spectroscopy of PDIC‐AC in H_2_O, no significant changes in peak shape were observed in PBS containing 10% FBS within 12 h, which suggests the excellent stability of PDIC‐AC under physiological conditions. Upon adding PDIC‐AC to PBS + 10% FBS, no new signal peaks were found compared to those in PBS + 10% FBS detected by DLS (Figure S9C). This presents additional evidence for the stability of PDIC‐AC in cell media. Combining these results with pKa data suggests that PDIC‐BH^+^ and DCA^−^ in PDIC‐AC undergo transient dissociation and binding processes under physiological conditions.

### 2.2 PDIs Inhibit Glycolysis by Directly Suppressing the Activity of PDHK1

The Cancer Genome Atlas (TCGA) database reveals that PDHK1 is upregulated in non‐small cell lung cancer (NSCLC) (Figure S10). Given that a PDHK inhibitor (DCA group) was incorporated into the perylene skeleton (Figure 1A), we thus focused our subsequent research on NSCLC, with an emphasis on metabolism and immunity mediated by PDIs. The inhibitory effect of PDIC‐NAC and PDIC‐AC on lung‐related tumor cells including human non‐small cell lung cancer cell A549 and mouse non‐small cell lung cancer cell LLC was firstly assessed by employing the standard 3‐(4, 5‐dimethylthiazole‐2‐yl)‐2, 5‐diphenyltetrazolium bromide (MTT) assay, and DCA was used as a comparative control in this work. The half maximal inhibition concentration (IC_50_) of PDIC‐AC on LLC cells was 0.39 µm, which was decreased significantly compared to DCA (12.35 mm), demonstrating that integrating DCA into perylene by ionic bond can effectively compensate for the weak antitumor activity of DCA. More importantly, PDIC‐AC also shows more potent killing efficiency on LLC cells than PDIC‐NAC (148.49 µm), which presents indubitable evidence for chemical bond modulated cytotoxicity. Moreover, the higher IC_50_ value against normal mouse murine lung epithelial cell MLE‐12 cells (3.84 µm) suggest stronger toxicity profile of PDIC‐AC toward cancer cells (Figure 1D and Figure S11). These notable variation in cell viability of PDIC‐AC and PDIC‐NAC prompted us to further investigate the underlying biological mechanism as discussion below.

As a crucial regulator of the pyruvate dehydrogenase complex (PDC), the inhibition of PDHK1 can enhance PDC activity, and thus shifts cellular metabolism from glycolysis towards mitochondrial OXPHOS (Figure 1E) [44, 45]. Reported work revealed that there are four action sites to reach PDHK1 inhibition, i.e., pyruvate‐binding site, CoA‐binding site, lipoamide‐binding site and ATP‐binding site [45]. As discussed above, PDIC‐AC and PDIC‐NAC exhibit distinct molecular scaffold and electron distribution, which is anticipated to affect the binding efficacy with the above action domain (Figure 1C and Figure S12). As expected, molecular docking simulations exhibited that PDIC‐NAC binds to the lipoamide site and forms an intermolecular hydrogen bond with Glutamine 61 (GLN‐61) with binding energy of ‐6.55 kcal mol^−1^ (Figure 1H). Given the ionic properties of PDIC‐AC, molecular docking simulations were respectively performed for PDIC‐BH^+^ and DCA^−^ with PDHK1. The results revealed that PDIC‐BH^+^ can bind to the lipoamide site and interact with GLN‐61 and Serine 75 (SER‐75) (binding energy: ‐7.10 kcal mol^−1^), while DCA^−^ occupies the pyruvate binding site with a binding energy of ‐1.61 kcal mol^−1^, suggesting a dual inhibitory on PDHK1 of PDIC‐AC (Figure 1H). This intriguing binding profile prompted us to investigate the direct binding of PDIs to PDHK1 via cellular thermal shift assay (CETSA). As illustrated in Figure 1F,G, PDHK1 kinase exhibited a thermal melting point (*T*m) of 50.2°C in PBS group. By comparison, PDIC‐AC possesses the *T*m of 55.7°C (Δ*T*m = +5.5°C), which is higher than that of PDIC‐NAC group (53.0°C), indicating the stronger binding interaction between PDIC‐AC and PDHK1. Given the conserved nature of the PDK family kinase domains, we hypothesized that PDIC‐BH^+^ might also interact with other isoforms. Both molecular docking and CETSA results demonstrated that PDIC‐BH^+^ exhibits binding affinity toward multiple PDHK isoforms (Figure S13). In line with the emphasis of this work on the crosstalk between energy metabolism and immune regulation, therefore, we focused our subsequently research efforts on PDHK1, a key regulatory protein that dominates glycolysis.

To further validate the interaction between PDIC‐AC and PDHK1 at specific residues (Ser‐75, Gln‐61), we used site‐directed mutagenesis to create expression plasmids in which the SER‐75 and GLN‐61 residues of PDHK1 were mutated to alanine (PDHK1‐MUT‐Q61‐S75A), with a Flag tag sequence introduced at the C‐terminus (Figures S14 and S15). After transfecting wild‐type (WT) and mutant (MUT) PDHK1 plasmids into cells for 60 h, the cells were lysed and then incubated with PDIC‐AC to assess binding ability. The CETSA results showed that the thermal stability of Flag tagged MUT‐PDHK1 (*T*m value increased from 49.8°C to 51.0°C) was significantly reduced after incubation with PDIC‐AC compared with Flag tagged WT‐PDHK1 (*T*m value increased from 50.3°C to 56.8°C) (Figure 1I and Figure S16). This indicates a decreased binding affinity between PDIC‐AC and MUT PDHK1, confirming that the SER‐75 and GLN‐61 residues may serve as binding sites of PDIC‐AC to PDHK1.

The enhanced binding interaction most likely exerts stronger inhibition effect on PDHK1 enzyme activity. To validate our hypothesis, PDHK1 enzyme activity was examined to probe the inhibitory effects of DCA, PDIC‐AC, and PDIC‐NAC on PDHK1 using an enzyme activity kit. To our surprise, PDIC‐AC achieved the most overwhelming inhibition effect on PDHK1 with the half maximal effective concertation (EC_50_) of 0.27 µM relative to DCA (76.67 µM) and PDIC‐NAC (39.19 µM) (Figure 1J and Figure S17). This dramatic enhancement in inhibitory efficacy confirms PDIC‐AC can function as a highly promising PDHK1 inhibitor for targeting therapy on PDHK1‐overexpressing cancers, and meanwhile highlights the significant advantage conferred by the ionic bond strategy.

PDHK1 inhibition is expected to activate PDC by reducing the phosphorylation of E1α subunit (p‐PDHA1) to further suppress the LA production [45, 46]. As anticipated, compared to PBS group, PDIC‐AC treatment can lead to the significant suppression of p‐PDHA1 level by 40% along with the decrease of intracellular LA by 61.47% in wild type (WT) LLC cells, which was substantially greater than that caused by PDIC‐NAC (20%, 46.38%) or DCA (20%, 35.17%) (Figure 1K,L). To determine whether the suppression of p‐PDHA1 and the reduction of LA is highly associated with PDHK1, we further transfected PDHK1‐specific siRNA plasmids into WT LLC cells (WT LLC) to construct a PDHK1‐knockdown LLC cell line (KD LLC). Knockdown efficiencies of approximately 90% for PDHK1 mRNA and 95.7% for PDHK1 protein, compared with the negative control (NC) group, was examined by quantitative real‐time polymerase chain reaction (qPCR) and western blot (WB), respectively (Figure S18 and Table S1), confirming the success of the knockdown operation. Following treatment with PDIC‐AC in KD LLC cells, p‐PDHA1 and LA level decreased by 20% and 55.81%, respectively, compared to the PBS group, which is a much smaller decline than that observed in WT LLC cells (40% and 61.47%) (Figure 1K,L). These comparative data strongly supported that PDIC‐AC initiate PDHK1‐dependent suppression on glycolysis in LLC cells. The extracellular acidification rate (ECAR) serves as a key indicator of glycolytic flux, reflecting the metabolic characteristics of tumor cells [46]. Seahorse XF Analyzer assays demonstrated that all compounds reduced ECAR in LLC cells to varying degrees. Notably, PDIC‐AC treated cells manifested a decrease of approximately 32% compared with PBS group, significantly greater than that in PDIC‐NAC (23.9%) and DCA groups (19.3%), presenting more vigorous evidence for substantial inhibition of glycolysis in LLC cells (Figure 1M,N).

### 2.3 PDIs Facilitate M1 Polarization of Tumor‐Associated Macrophages (TAMs) via Direct PDHK1 Inhibition

**FIGURE 2:**
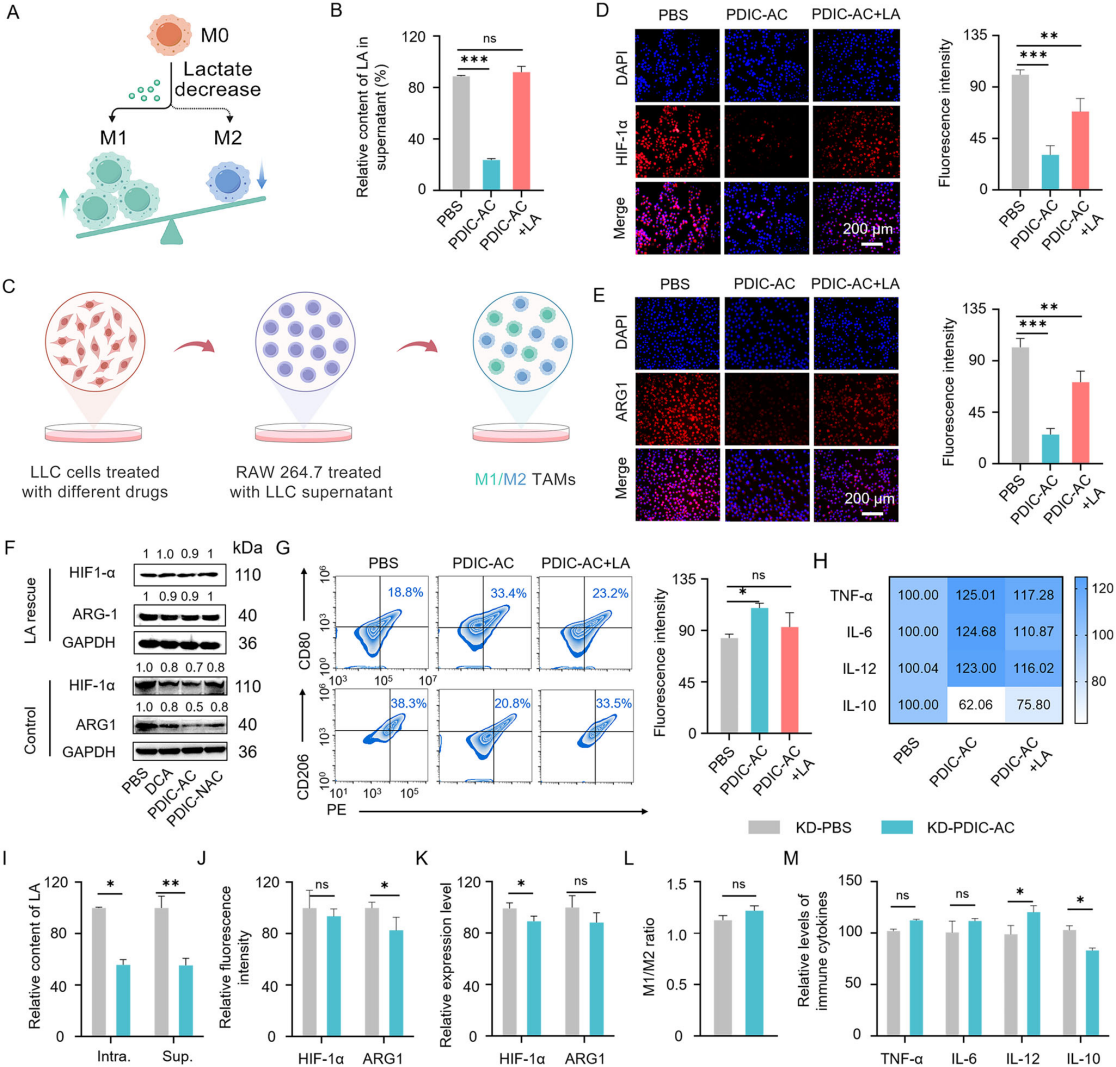
PDIC‐AC reprograms immunosuppression by suppressing LA secretion. (A) Schematic diagram of the macrophage polarization towards M1 phenotype by suppressing LA secretion. (B) Relative levels of LA in the supernatant of LLC cells following treatment with PDIC‐AC and LA supplementation. (C) Schematic diagram of PDIs‐induced macrophage differentiation. Immunofluorescence and quantitative results of (D) HIF‐1α and (E) ARG1 in RAW 264.7 after treatment by conditioned medium and statistical results. (F) Intracellular HIF‐1α and ARG1 expression level in RAW 264.7 after treatment by conditioned medium. (G) M1 or M2 macrophage phenotyping of RAW 264.7 upon PDIC‐AC or PDIC‐AC+LA treatment. (H) IL‐6, IL‐10, TNF‐α and IL‐12 cytokines in conditioned medium after cultured RAW 264.7. (I) The relative LA levels in the intracellular (Intra.) and supernatant (Sup.) in KD LLC cells after treatment with PDIC‐AC. The quantification results of (J) immunofluorescence, (K) HIF‐1α and ARG1 in RAW 264.7, and (L) M1/M2 ratio after treatment with conditional medium collected from KD LLC cells. (M) IL‐6, IL‐10, TNF‐α and IL‐12 cytokines in TAMs incubated with KD LLC cell supernatant. Data represent mean ± SD (*n* = 3). Statistical comparisons were performed using one‐way ANOVA. ns, not significant (*p* > 0.05); *p < 0.05; **p < 0.01; ***p < 0.001.

TAMs, broadly classified into M1 and M2 subtypes, play a pivotal role in orchestrating tumor immunosuppression and immune evasion [47]. M1‐type TAMs are instrumental in antigen presentation and initiating an immune response against invading pathogens. Conversely, M2‐type TAMs are renowned for their immunosuppressive capabilities. In established solid tumors, most TAMs exhibit M2‐like phenotype, serving as key accomplices in the tumor microenvironment to achieve immune suppression and promote tumor growth [48, 49]. LA, a crucial mediator of glycolytic metabolism, has emerged as a significant modulator of macrophage phenotypic plasticity (Figure 2A). In the tumor microenvironment, reducing LA levels can alleviate the inhibition of the hypoxia‐inducible factor alpha (HIF‐1α) signaling pathway and promote the polarization of M2 macrophages towards M1 ones [50].

PDIs robustly inhibited LA production in LLC cells, resulting in a 73.18% reduction of LA in the cell supernatant (Figure 2B and Table S2), we further evaluated the HIF‐1α and the arginase‐1 (ARG1, one marker for M2‐type TAMs) expression level in RAW 264.7 cells by immunofluorescence and WB assays. As depicted in Figure 2C, LLC cells were pretreated with PBS, DCA, PDIC‐AC and PDIC‐NAC for 12 h, and then the medium was replaced with fresh one and cultured for another 12 h. The resulting conditioned supernatant was collected and applied to incubate undifferentiated murine macrophage RAW 264.7 cells (M0) to conduct polarized macrophage. Taking PDIC‐AC group as an example, the fluorescence intensity of HIF‐1α and ARG1 were decreased to 30.91% and 25.64% after incubated with conditioned medium, compared to PBS group (Figure 2D,E), and these results were consistently supported by WB data (Figure 2F). Moreover, flow cytometry analysis indicates that, M1/M2 macrophage yielding a ratio of 0.49 after incubated with conditioned medium from PBS group, indicative of a dominant immunosuppressive phenotype. The administration of PDIC‐AC altered the M1/M2 ratio to 1.59, indicating that PDIC‐AC significantly arouse the preponderance of immune‐promoting macrophages (Figure 2G). PDIC‐AC effectively upregulates the secretion of pro‐inflammatory cytokines by M1‐type macrophages. Specifically, compared to the PBS group, there was an increase of 25.01% for tumor necrosis factor‐α (TNF‐α), 24.68% for interleukin (IL)‐6, and 23.00% for IL‐12. Meanwhile, there was a 37.94% decrease in the anti‐inflammatory cytokine IL‐10, which is secreted by M2‐type macrophages (Figure 2H). A similar regulation in macrophage polarization was also achieved for DCA and PDIC‐NAC groups (Figures S19–S23). These results confirmed that treatment with conditioned medium collected from PDI treatment group can efficiently promote the polarization of macrophages from M2 phenotype to M1 phenotype.

To further verify whether LA mediates the observed immunomodulatory effects on macrophages, we performed LA rescue experiment by supplementing exogenous LA into the conditional medium in PDIC‐AC group (termed as PDIC‐AC+LA group) and achieve the same concentration as the PBS group (Figure 2B and Figure S23). As shown in Figure 2D,E, the expression levels of HIF‐1α and ARG1 in PDIC‐AC+LA group restored to 68.83% and 71.34% of that in PDIC‐AC group, respectively, and it is further validated by WB results (Figure 2F). Meanwhile, the M1/M2 ratio was shifted from 1.38 in PDIC‐AC group into 1.02 in PDIC‐AC+LA group (Figure 2G), implying that LA is involved in the process of macrophage polarization induced by PDIC‐AC. In addition, compared to PDIC‐AC group, PDIC‐AC+LA effectively added back the pro‐inflammatory cytokines secreted by M1‐type macrophages (7.73% for TNF‐α, 13.81% for IL‐6 and 6.98% for IL‐12), and meanwhile added back the anti‐inflammatory cytokine IL‐10 by 13.2% (Figure 2H). These results suggested that exogenous LA supplementation decreased the secretion of pro‐inflammatory cytokines and increased the secretion of anti‐inflammatory cytokines by macrophages, further confirming that LA influences the polarization from M2 towards the M1 phenotype.

To verify whether the action of PDIs on macrophage polarization depends heavily on PDHK1 activity, we further evaluated these results in RAW 264.7 cells incubated in KD LLC cell conditioned medium. Taking PDIC‐AC group as an example, the results demonstrate that the relative LA level in intracellular and in the supernatant of KD LLC cells was suppressed by 44.19% and 44.51% along with the decrease of the HIF‐1α and ARG1 expression level in RAW 264.7 cells by 17.28% and 6.37%, respectively (Figure 2I–K; Figures S24–S26 and Tables S2 and S3). Flow cytometry analysis of RAW264.7 cell polarization revealed that the minor increase (10%) of M1/M2 ratio was reached in KD LLC cell, which is significantly lower than WT LLC group (224.5%) (Figure 2L; Figure S27 and Table S4). Consistently, PDIC‐AC treatment just induced a modest upregulation of the pro‐inflammatory cytokines TNF‐α, IL‐6, and IL‐12, while concurrently causing a slight reduction in the secretion of the anti‐inflammatory cytokine IL‐10, compared to PBS group (Figure 2M; Figure S28 and Table S5). The above findings demonstrated that PDHK1 knockdown abolished the capacity of PDIC‐AC to reduce LA production and its associated effects, definitively establishing that PDIC‐AC acts via the PDHK1‐PDC axis to alleviate immunosuppression in the tumor microenvironment, which highlighting its potential for cancer immunotherapy.

Collectively, these findings establish a clear causal link—PDIC‐AC inhibits PDHK1 activity and suppresses LA production, which in turn drives macrophage polarization toward the M1 phenotype and modulates cytokine secretion. LA supplementation experiments further confirm that exogenous LA effectively reverses this process, thereby functionally validating the causal relationship between metabolic inhibition, LA reduction, and immune improvement.

### 2.4 PDIs Trigger Endogenous ROS Production via Reducing Substances or Electron Transport Chain (ETC) Imbalance

**FIGURE 3:**
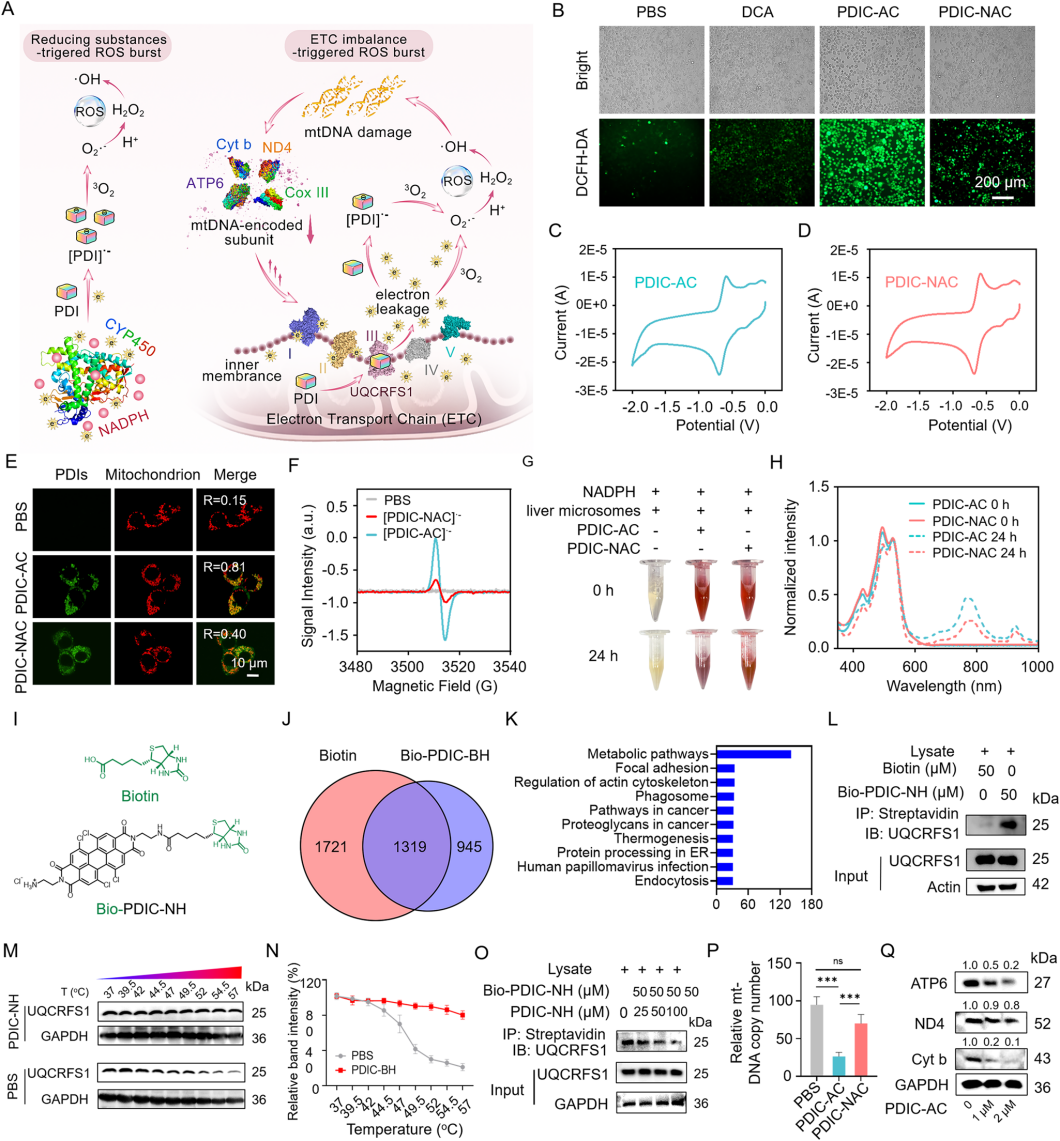
PDIs‐mediated endogenous ROS production. (A) Schematic diagram of ROS generation mechanism by PDIs. (B) Representative fluorescence images of total ROS in LLC cells after incubation of DCA, PDIC‐AC or PDIC‐NAC for 8 h. Cyclic voltammetry of the reversible reduction of (C) PDIC‐AC and (D) PDIC‐NAC at scan rate of 20 mV s^−^^1^. (E) Representative images of mitochondrial colocalization of PDIC‐AC and PDIC‐NAC. (F) EPR spectra of [PDIC‐AC]^•−^ and [PDIC‐NAC]^•−^ in LLC cells. (G) Color and (H) UV–Vis of [PDIC‐AC]^•−^ and [PDIC‐NAC]^•−^ in the NADPH/liver microsomal system. (I) The molecular structures of Biotin, PDIC‐BH and Bio‐PDIC‐NH. (J) Venn diagram of proteins identified by mass spectrometry. (K) Top 10 specific enriched KEGG pathways of Bio‐PDIC‐NH interacting proteins. (L) IB and IP analysis of the streptavidin‐agarose precipitated UQCRFS1 from LLC cells incubated with Bio‐PDIC‐NH. (M) CETSA and (N) quantitative results of UQCRFS1 treated by PDIC‐NH in LLC cells. (O) Competitive binding UQCRFS1 between PDIC‐BH and Bio‐PDIC‐NH. (P) mtDNA copy number in LLC cells after treated with PDIC‐AC or PDIC‐NAC for 12 h by qPCR. mtDNA were normalized to β‐actin mRNA encoded by the nuclear gene. (Q) The expression levels of mtDNA encoded mitochondrial complexes. Data are shown as mean ± SD (*n* = 3). Statistical comparisons were performed using one‐way ANOVA. ns, not significant (*p* > 0.05); ***p < 0.001.

It is well known that mitochondria is an essential place for endogenous ROS (i.e., O_2_^•−^, H_2_O_2_ and ^•^OH) production [51]. For one thing, destruction of PDIs on ETC complexes in mitochondria can cause the electron leakage, and these electrons can directly transfer to triplet oxygen (^3^O_2_) to promote the production of O_2_^•−^, which can undergo dismutation and Fenton‐type reaction to produce H_2_O_2_ and ^•^OH (Figure 3A). For another thing, given the highly electron deficient structural characteristics of PDIs, it is proposed that mitochondria‐distributed PDIs will obtain these leaked electrons to be reduced into delocalized radical anions ([PDI]^•−^), and then ^3^O_2_ may capture electrons from [PDI]^•−^ to promote the production of endogenous ROS. Additionally, PDI has been confirmed to capture electrons from reductive substances to generate [PDI]^•−^ in hypoxic tumor cell [37], which, being different from ETC imbalance pathway, can function as the reductive substances channel to promote the endogenous ROS production (Figure 3A). It should be pointed that there is a competition between PDI or ^3^O_2_ to acquire electrons in mitochondria. The competition is highly determined by their initial reducing potential. The lower the reducing potential, the greater ability to obtain electrons [52].

Given these facts, we take PDIC‐AC and PDIC‐NAC as an example to elaborate possible mechanism for PDI‐triggered endogenous ROS burst. As evidenced in Figure 3B and Figure S29, the fluorescence intense in PDIC‐AC group is 8.88 times than that in PDIC‐NAC group, clearly demonstrating superior ROS generation capability of PDIC‐AC, and this striking enhancement was definitively established by flow cytometry. Specifically, the yields of *O*_2_^· −^, H_2_O_2_, and ^•^OH triggered by PDIC‐AC were 3.83, 4.60, and 3.2 folds higher than those in the PDIC‐NAC group (Figures S30–S32), respectively. These results collectively indicate that the introduction of ionic bond markedly enhances ROS generation capacity. Cyclic voltammetry revealed that PDIC‐AC exhibited a slightly lower initial reduction potential (‐0.086 V) compared to PDIC‐NAC (‐0.098 V) (Figure 3C,D), but both of which are substantially lower than that of oxygen (‐0.33 V, vs SHE), suggesting that ROS burst mainly depended on the formation of both [PDIC‐AC]^•−^ and [PDIC‐NAC]^•−^ rather than direct electron transfer to ^3^O_2_ [53, 54]. Furthermore, mitochondrial co‐localization imaging revealed PDIC‐AC exhibited a markedly higher Pearson's coefficient (0.85) compared to PDIC‐NAC (0.42) (Figure 3E), implying that PDIC‐AC may mainly obtain electron from ETC in mitochondria, while PDIC‐NAC primarily capture electrons from the reductive substances in cytoplasm. Based on this finding, we further employed electron paramagnetic resonance (EPR) spectroscopy to detect the generation of [PDI]^•−^ in LLC cells. As illustrated in Figure 3F, both samples exhibited characteristic radical signals at *g* = 2.0034, further confirming that PDIC‐AC and PDIC‐NAC can be reduced to form [PDIC‐AC]^•−^ or [PDIC‐NAC]^•−^. The higher EPR peak intensity implies the superior generation ability of [PDIC‐AC]^•−^ than [PDIC‐NAC]^•−^, presenting an indubitable evidence for more efficient ROS production in PDIC‐AC group than that in PDIC‐NAC group.

Based on the above hypothesis and findings, we first explored the generation mechanism of [PDI]^•−^ in the cytoplasm using PDIC‐NAC as an example. Previous studies have reported that PDI can capture electrons from reducing substances to form [PDI]^•−^ in hypoxic tumor cells, it does not clarify specific reducing substances to verify this reductive process [37, 41, 42]. In light of this limitation, we firstly investigate the possible reducing substances that can induce the formation of [PDIC‑NAC]^•−^ in vitro. Tumor cells harbor various reducing substances that can act as electron donors. The most prominent of these are nicotinamide adenine dinucleotide phosphate hydrogen (NADPH), NADH, glutathione (GSH), thioredoxin (Trx), vitamin C, and α‐lipoic acid [55, 56]. In biological systems, NADPH serves as a universal electron donor for various oxidoreductases, including cytochrome P450 (CYP450) reductase, NADPH dehydrogenase, monooxygenases, and GSH reductase, facilitates electron transfer [57, 58]. Against the background of the above research, we designed diverse systems to explore the specific participants that reduced PDIC‐NAC into [PDIC‐NAC]^•−^. It is found that when liver microsomes (the most commonly used in vitro model for studying CYP450 enzyme function), NADPH and PDIC‐NAC were co‐incubated in PBS at 37°C for 24 h, the color of PDIC‐NAC changed from bright red to light purple (Figure 3G). This change was accompanied by the emergence of characteristic absorption peaks of [PDIC‐NAC]^•−^ at approximately 770 and 924 nm (Figure 3H) [37], validating that NADPH/liver microsome can act as the electron donor to initiate the formation of [PDIC‐NAC]^•−^.

We then use PDIC‐AC as an example to investigate the possible production mechanism of [PDI]^•−^ in mitochondria. It is speculated that PDIC‐AC binds to mitochondrial complexes and interferes with the ETC to cause electron leakage. To this end, we synthesized a biotin‐labeled perylene probe, Bio‐PDIC‐NH, via amidation, and employed it in pull‐down assays to capture interacting proteins from cell lysates (Figure 3I). Mass spectrometry identified 945 proteins that specifically bound to Bio‐PDIC‐NH among a total of 3,985 proteins (Figure 3J). Kyoto Encyclopedia of Genes and Genomes (KEGG) analysis revealed that metabolic pathways were the most significantly enriched category (Figure 3K), encompassing multiple mitochondrial complex proteins such as UQCRFS1, ubiquinone oxidoreductase subunit B10 (NDUFB10) and ubiquinone oxidoreductase core subunit S3 (NDUFS3). Furthermore, immunoprecipitation (IP) was performed using streptavidin magnetic beads, followed by immunoblotting (IB) with an antibody against UQCRFS1. The results revealed that Bio‐PDIC‐BH pulled down the specific Complex III subunit UQCRFS1 more effectively than the Biotin group did (Figure 3L). The robust interaction between Bio‐PDIC‐NH and UQCRFS1 was confirmed by the CETSA assay (Figure 3M,N). To determine the binding region of UQCRFS1 with Bio‐PDIC‐NH more precisely, we performed a competitive binding assay. Briefly, cell lysates were pre‐incubated with increasing concentrations of unlabeled PDIC‐NH (25, 50, and 100 µm) to competitively block protein binding sites. After adding Bio‐PDIC‐NH at a fixed concentration (50 µm) and incubating for 4 h at 4°C, the protein complexes were pulled down using streptavidin‐modified magnetic beads. Subsequent IP and IB analysis revealed that, compared with the Bio‐PDIC‐NH group, the amount of UQCRFS1 decreased in a dose‐dependent manner with increasing concentrations of free PDIC‐NH (Figure 3O). This indicates that UQCRFS1 specifically binds to the PDI skeleton of Bio‐PDIC‐NH rather than the biotin group. UQCRFS1, the core catalytic subunit of mitochondrial complex III, is responsible for transferring electrons from ubiquinol to cytochrome c. Dysfunction of UQCRFS1 causes an imbalance in the ETC and electron leakage, and the leaked electrons are captured by PDIC‐AC to form [PDIC‐AC]^•−^, promoting endogenous ROS production. Furthermore, ROS burst can induce damage to mitochondrial DNA (mtDNA) (Figure 3P), which impairs the transcription and translation of mtDNA‐encoded complex subunits, including Cyt b, ND4, Cox III, and ATP6 (Figure 3Q and Figure S33). This exacerbates ETC dysfunction, establishing a cycle that promotes electron leakage and ROS burst.

Based on these findings, it is evident that PDIs can stimulate the production of endogenous ROS in cells through reducing substances or ETC imbalance. The efficiency of ROS production depends heavily on the quantity of [PDI]^•−^ formed, which is determined by its initial reduction potential and cell distribution. Additionally, we verified the specific electron source for [PDI]^•−^ generation in both the cytoplasm and mitochondria. To the best of our knowledge, this is the first study to establish an unambiguous mechanism for PDI‐triggered endogenous ROS generation in hypoxic cells.

### 2.5 PDIC‐AC Triggers ICD by Impairing OXPHOS

**FIGURE 4:**
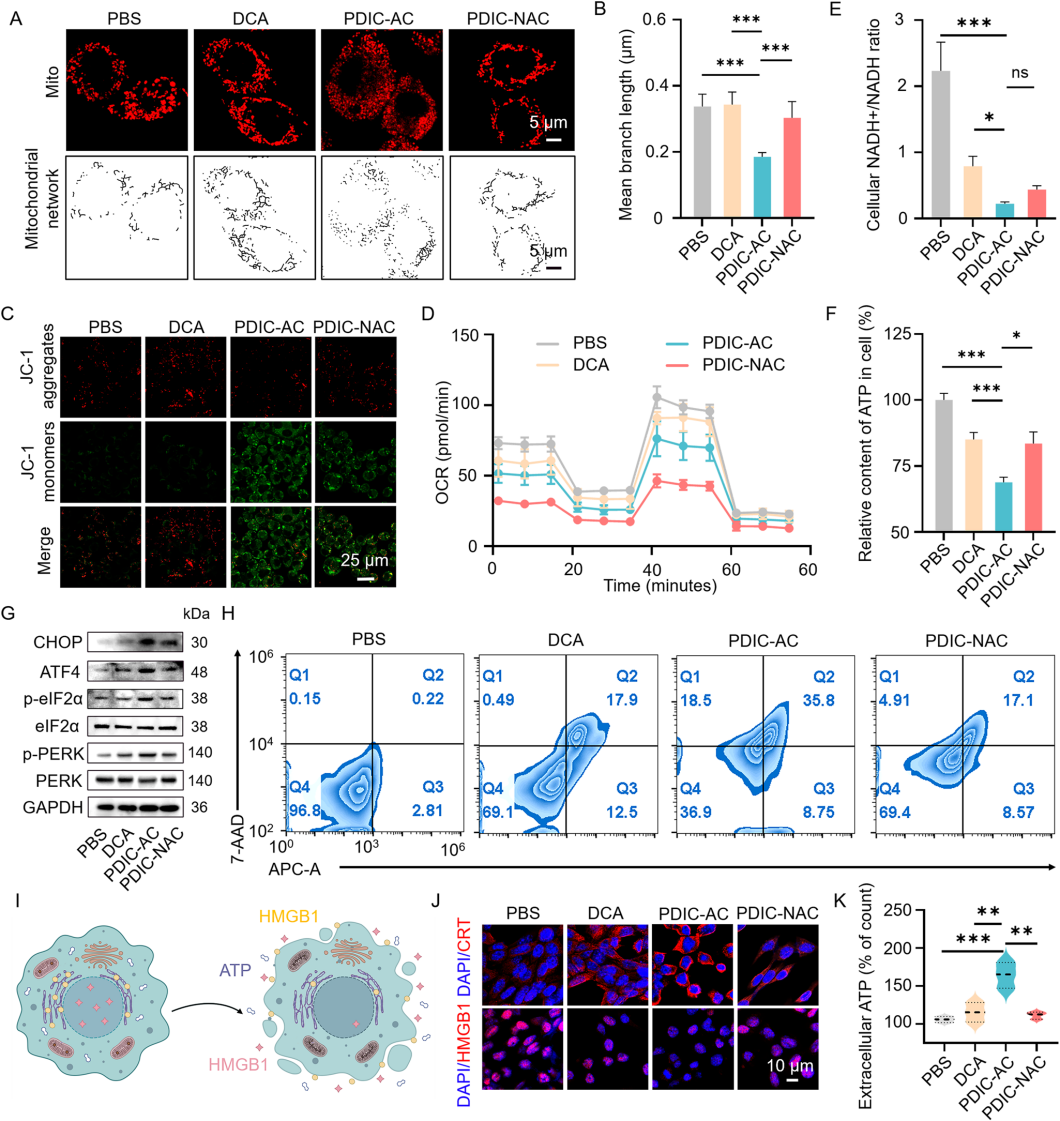
PDIs disrupt OXPHOS to trigger ICD effects. (A) Fluorescence images of LLC cell labeled with Mito‐Tracker Red CMXRos and (B) quantitative result of mean branch length in mitochondrial networks in Figure 4A. (C) Representative fluorescence images of JC‐1 aggregates/JC‐1 monomers in LLC cells. (D) OCR measured by Seahorse Mito Stress Test in LLC cells treated with DCA, PDIC‑AC and PDIC‑NAC. (E) Intracellular NAD+/NADH ratio in LLC cells treated by DCA, PDIC‐AC and PDIC‐NAC. (F) Intracellular ATP content in LLC cells after different treatment. (G) Representative proteins associated with ER stress signaling pathway. (H) Apoptosis ration of LLC cell examined by flow cytometry. (I) Schematic diagram for ICD effect. (J) Representative immunofluorescence images of the externalization of CRT (red) and the release of HMGB1 (red) of LLC cells. (K) The intracellular ATP content in LLC cells. Data represent mean ± SD (*n* = 3). Statistical comparisons were performed using one‐way ANOVA. ns, not significant (*p* > 0.05); *p < 0.05; **p < 0.01; ***p < 0.001.

It is well‐established that excessive ROS can disrupt OXPHOS, which can severely impair mitochondrial function and morphology. Prompted by this connection, we firstly investigated the effects of DCA, PDIC‐AC and PDIC‐NAC on mitochondrial morphology in LLC cells. As observed in mitochondrial fluorescence images marked with Mito‐Tracker Red CMXRos (a mitochondrial membrane potential‐dependent dye) (Figure 4A), mitochondria in PBS group appeared as numerous clear scattered, punctate, or short rod‐shaped structures. In contrast, cells treated with DCA and PDIC‐NAC predominantly exhibited elongated tubular mitochondria, which tended to fuse with each other. Notably, PDIC‐AC group showed punctate or short rod‐shaped mitochondria with blurred boundaries and diminished, diffused dye fluorescence (Figure 4A), suggesting the serious damage of mitochondrial structure and morphology. The mitochondrial morphometric quantification acquired by Image J show that the complexity of the mitochondrial network increased in both DCA and PDIC‐NAC groups (Figure 4A). However, PDIC‐AC treatment reduced mitochondrial complexity in LLC cells, which was evidenced by a decrease in the average mitochondrial branch length to 54.84% and 61.03% of that in PBS (0.34 µm) and PDIC‐NAC (0.18 µm) groups, respectively (Figure 4B), supporting that PDIC‐AC impaired mitochondrial integrity. Building upon the observed mitochondrial network fragmentation and impaired dye retention, we further assess mitochondrial function by measuring the membrane potential (Δ*Ψ*m). As shown in Figure 4C and Figure S34, JC‐1 probe detection revealed bright red fluorescence in PBS group, indicating intact mitochondrial membrane potential. Following PDIC‐AC treatment, Δ*Ψ*m profoundly impaired and reduced to 8.16% of PBS group. Furthermore, the cellular energy metabolism assay indicated that PDIC‐AC treatment drastically reduced OCR to 40.7% of the PBS group (Figure 4D and Figure S35) These results collectively demonstrated that PDIC‐AC severely disrupted OXPHOS process in LLC cells.

As stated above, PDIC‐AC intervention instigates efficient inhibition of glycolysis and OXPHOS, which not only curtails nicotinamide adenine dinucleotide (NAD+/NADH) conversion, but also suppresses ATP production (Figure 1E) [59, 60]. As shown in Figure 4E, the NAD+/NADH ratio in PDIC‐AC group decreased to 0.22, which was only 10.0% of that in PBS group. Correspondingly, the level of ATP secretion in LLC cells of the PDIC‐AC group was 68.82% of that in PBS group (Figure 4F). The results demonstrated that the inhibition of glycolysis and OXPHOS could disrupt the NAD+/NADH cycle and suppress ATP production effectively.

Concurrently, excessive ROS can disrupt cellular redox homeostasis, thereby initiating cellular stress programmer and activating downstream response pathway. Specially, ROS oxidatively modifies the PKR‐like endoplasmic reticulum kinase (PERK) and triggers its auto‐phosphorylation. Subsequently, phosphorylated PERK (p‐PERK) targets eukaryotic translation initiation factor 2α (eIF2α) and induces its phosphorylation (p‐elF2α), which selectively promotes the translation of activating transcription factor 4 (ATF4). The upregulation of ATF4 strongly induces the expression of its key target gene C/EBP‐homologous protein (CHOP). As shown in Figure 4G, following treatment with PDIC‐AC, the relative expression levels of p‐PERK, p‐eIF2α, ATF4 was increased by 20%, 200% and 60%, compared to PBS group, validating that PDIC‐AC effectively activates PERK‐eIF2α‐ATF4‐CHOP oxidative stress pathway.

ATP suppression and oxidative stress can simultaneously induce apoptosis of tumor cells [54]. Flow cytometry analysis demonstrated that PDIC‐AC and PDIC‐NAC treatment induced apoptosis rates of 44.55% and 25.67%, respectively (Figure 4H and Figure S36). To further verify the causal relationship between energy depletion and cell apoptosis, we performed rescue experiments with exogenous ATP and methyl pyruvate. Specially, PDIC‐AC‐treated LLC cells were co‐incubated with exogenous ATP (1 mm) or methyl pyruvate (1 mm) for 12 h, and subsequently detected via flow cytometry. As presented in Figure S37, co‐treatment of methyl pyruvate failed to rescue PDIC‐AC‐induced apoptosis, which indicates that PDIC‐AC may exert its cytotoxic effects through direct impairment of mitochondrial function or activation of other non‐energy‐metabolism‐related death signaling pathways. To note, exogenous ATP supplementation markedly reduced the apoptotic rate to 28.4%, demonstrating a partial reversal of PDIC‐AC‐elicited cell death, providing strong evidence that energy depletion acts as a critical driver of apoptosis triggered by PDIC‐AC. WB analysis revealed that following PDIC‐AC treatment, the expression level of the anti‐apoptotic protein Bcl‐2 level decreased by 50% compared to PBS group, and it was 5 times higher than that in PDIC‐NAC group (Figure S38). The pro‐apoptotic protein Bax in PDIC‐AC group was upregulated by 40% than PBS and PDIC‐NAC, respectively. Cleaved caspase‐3 is one of the most critical markers of cell apoptosis, and its activation will lead to irreversible cell death. As shown in Figure S38, the expression level of cleaved caspase‐3 in PDIC‐AC group was increased by 30% and 10% than PBS and PDIC‐NAC groups, respectively. These results indicate that PDIC‐AC effectively promotes tumor cell apoptosis by inhibiting ATP production and inducing oxidative stress.

Concurrently, the activation of the PERK‑eIF2α‑ATF4‑CHOP signaling pathway can trigger immunogenic cell death (ICD) in tumor cells, thereby sending “danger” signals to the immune system and eliciting specific anti‑tumor immune responses (Figure 4I). To further characterize this process, we assessed the release of damage‑associated molecular patterns (DAMPs), including calreticulin (CRT), ATP, and high‑mobility group box 1 protein (HMGB1). Compared with PBS, DCA and PDIC‐NAC groups, PDIC‐AC treated cells exhibited CRT translocation to the cell membrane (Figure 4J), with extracellular ATP release levels reaching 6.5 and 3.4 folds of that in PBS and PDIC‐NAC group, respectively (Figure 4K). While HMGB1 fluorescence intensity decreased to 40% and 80% of the levels observed in PBS and PDIC‐NAC group, respectively (Figure 4J). Additionally, compared with 1 µm or 4 µm PDIC‑AC, treatment with 2 µm PDIC‑AC led to markedly enhanced CRT exposure, HMGB1 release, and ATP efflux (Figure 4J and Figure S39). To this end, we subsequently generated an LLC‑based immune vaccine using PDIC‑AC (2 µm) and evaluated its efficacy in eliciting in vivo immune activation.

### 2.6 PDIC‐AC‐Induced ICD Effect Confers In Vivo Antitumor Protection

**FIGURE 5:**
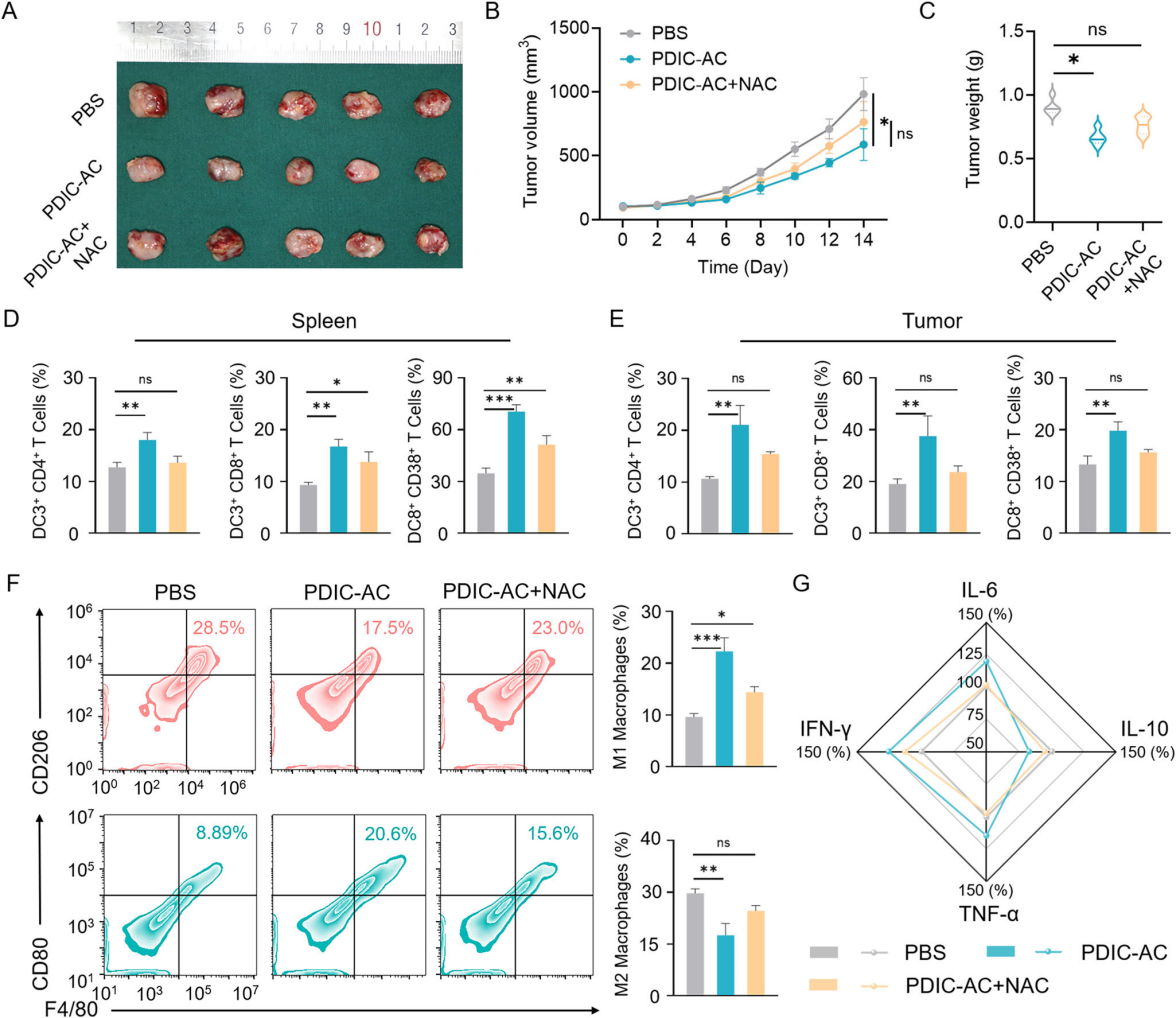
(A) Photographs of tumor tissues in the different groups at the end of the treatment, (B) tumor volume and (C) tumor weight. The helper T cells, CD8^+^ T cells and activated CD8^+^ T cells in (D) spleen and (E) tumor detected by flow cytometry. (F) Macrophage phenotyping and quantitative results. (G) IL‐6, IL‐10, TNF‐α and IL‐12 cytokines in serum. Data represent mean ± SD (*n* = 3). Statistical comparisons were performed using one‐way ANOVA. ns, not significant (*p* > 0.05); *p < 0.05; **p < 0.01; ***p < 0.001.

Given that ICD effects were effectively activated at the cellular level (Figure 4I–K), we subsequently constructed tumor vaccine to further validate in vivo ICD effect on immune activation and response. Specifically, LLC cells were firstly treated with PDIC‐AC (2 µm) or PBS for 12 h. Then, the cells were harvested, lysed and subcutaneously injected into normal C57BL/6N mice 7 d after vaccination, all mice were challenged with a subcutaneous injection of live LLC cells into the opposite flank. Tumor growth was monitored every 2 d thereafter to assess the protective immune response. Fourteen days later, all mice were euthanized and their subcutaneous tumors were carefully excised and photographed. As shown in Figure 5A, tumors in PDIC‐AC‐treated group exhibited slower growth than those in PBS group. Statistical analysis revealed that the tumor volume and weight in the PDIC‐AC‐treated group were 608.35 mm^3^ and 668.35 mg, respectively. These values were significantly lower than those in PBS group (882.6 mm^3^ and 904.01 mg) (Figure 5B,C), suggesting that PDIC‐AC‐induced ICD effect can effectively inhibit distant tumor progression.

We further validated the ICD effect on immune activation and response by evaluating dendritic cells (DCs) maturation, T cell activation, and macrophage polarization. As shown in Figure S40, the DCs maturation rate in PDIC‐AC group was 57.6%, which is 2.5 times that of PBS group. The maturation of DCs can activate T cells. The results in Figure 5D and Figure S40 showed that the proportions of helper T cells (CD3^+^CD4^+^ T cells), cytotoxic T cells (CD3^+^CD8^+^ T cells) and activated CD8^+^ T cells (CD3^+^CD8^+^CD38^+^ T cells) in the spleen of the PDIC‐AC group were 18.7%, 17.2% and 66.4%, respectively, which was 1.36, 1.87, and 2.04 times higher than that in PBS group. A similar activation ratio of T cells in the tumor was also detected (Figure 5E). Furthermore, PDIC‐AC treatment initiated the elevation of M1 macrophage ratio from 8.89% (PBS group) to 20.6%, while the M2 ratio decreased from 28.5% to 17.5% (Figure 5F), demonstrating that the immunotherapeutic vaccine successfully re‐educates macrophages within the tumor microenvironment from the pro‐tumor M2 phenotype to an anti‐tumor M1 phenotype. We further examined the secretion levels of cytokines in the serum of immunized mice. As shown in Figure 5G, in the PDIC‐AC group, secretion levels of the pro‐inflammatory cytokines TNF‐α, IFN‐γ, and IL‐6 increased by 14.97%, 33.17%, and 19.49%, respectively, while anti‐inflammatory cytokine IL‐10 level decreased by 13.4%, compared with PBS group, suggesting that the immune vaccine elicited an effective immune response. Overall, these results indicated that PDIC‐AC‐induced ICD effect can efficiently activate adaptive antitumor immune responses in mice.

To validate the contribution of ROS to therapeutic outcomes, we further constructed tumor vaccine by subcutaneously injected LLC cells under co‐treatment with PDIC‐AC and ROS scavenger N‐acetylcysteine (NAC). In contrast, PDIC‐AC+NAC treatment reversed the antitumor effect of PDIC‐AC, as evidenced by the significant increase in the mean tumor volume and weight (763.76 mm^3^ and 763.76 mg) in PDIC‐AC+NAC group than those in PDIC‐AC group (608.35 mm^3^ and 668.35 mg) (Figure 5A–C). This suggests that ROS play a critical role in mediating anti‐tumor efficacy, which is confirmed by T activation and the macrophage polarization. As shown in Figure 5D and Figure S41, the activation rates of helper T cells, CD8^+^ T cells, and activated CD8^+^ T cells in the spleen from PDIC‐AC+NAC group decreased by 14.2%, 13.6%, and 53.8%, respectively, compared to that of PDIC‐AC group, Additionally, the ratio of M1 macrophages decreased from 20.6% to 15.6% in PDIC‐AC group following NAC treatment, while the ratio of M2 macrophages increased from 17.5% to 23.0% (Figure 5F), further verified that ROS plays a key role in antitumor immune response triggered by PDIC‐AC.

### 2.7 Biocompatibility and Pharmacokinetic Properties of PDIs

Based on the in vitro significant biological activity and outstanding immune‐activating effects, we are highly encouraged to proceed with investigating the antitumor effects of PDIC‐AC and PDIC‐NAC in mice. Before that, we evaluated the biocompatibility of PDIC‐AC and PDIC‐NAC in C57BL/6N mice. As shown in Figure S42, the mice appeared 100% surviving state in 30‐day experimental process without weight loss at the dosage of 2, 5, and 10 mg kg^−1^ every three days. Blood routine/biochemical indicators and hematoxylin and eosin (H&E) staining indicate that there is no obvious damage to organs or the blood system (Figures S42 and S43), confirming that PDIC‐AC and PDIC‐NAC exhibit favorable biosafety upon the dosage up to 10 mg kg^−1^. Subsequently, we investigated the in vivo distribution and pharmacokinetic properties of PDIC‐AC. In vivo imaging studies demonstrated that PDIC‐AC mainly accumulates in lung tissue following intravenous administration (Figure S44), establishing a robust foundation for highly effective treatment of lung cancer. Additionally, the single‐dose pharmacokinetic profile revealed that PDIC‐AC has a half‐life (*T*_1/2_) of 9.32 h and a mean residence time (MRT_0‐inf_) of 13.13 h (Table S6), implying that PDIC‐AC possesses the long circulation time in blood to improve treatment efficiency.

### 2.8 Evaluation of Chemo‐Immunotherapy Efficacy on Primary Lung Tumor

**FIGURE 6:**
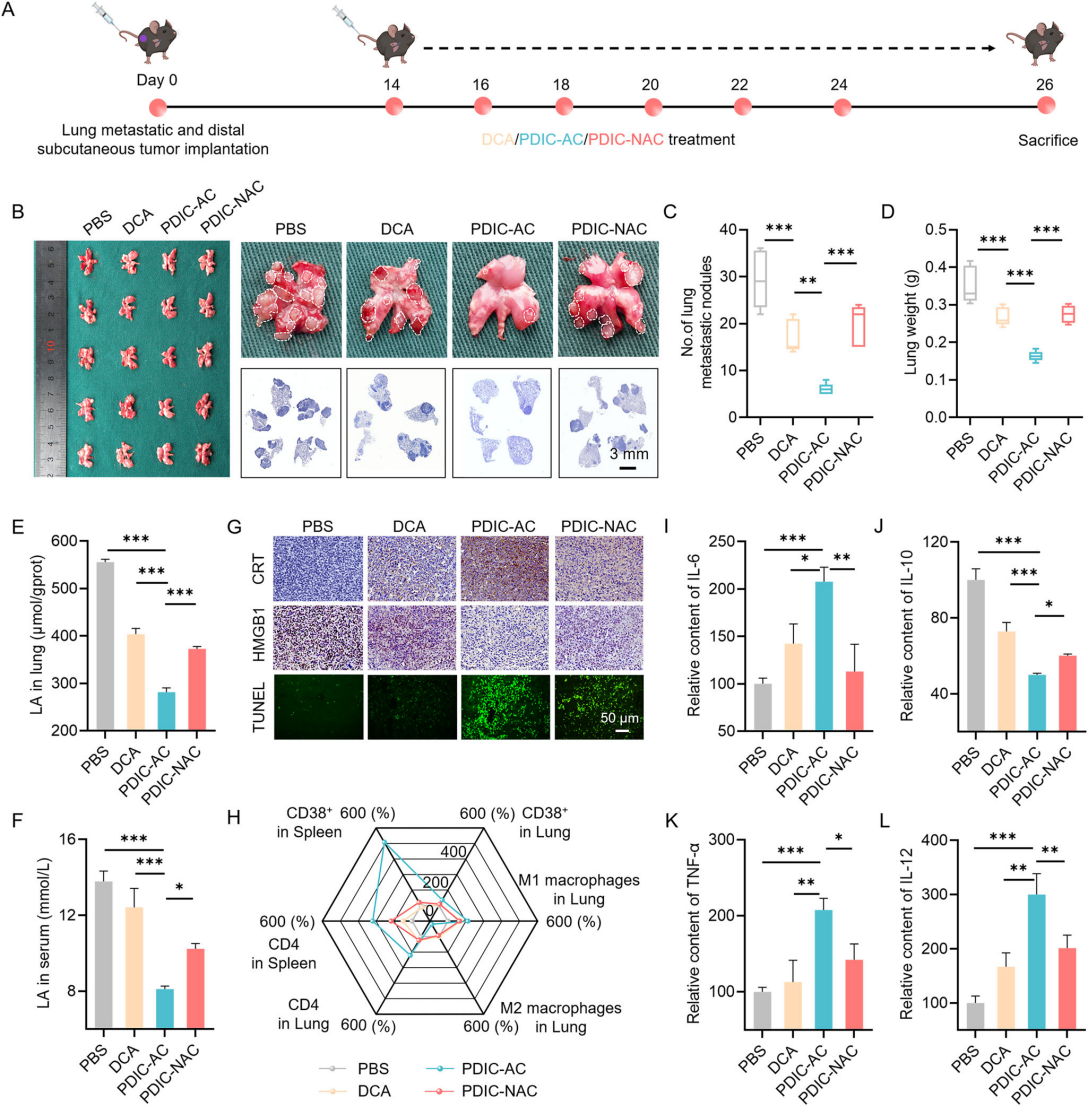
Evaluation of the chemo‐immunotherapy efficacy on primary lung tumor. (A) Schematic diagram of the establishment of lung cancer model and treatment protocol. (B) Photographs of lung tissues and magnified images of representative lungs as well as H&E staining. The area circled by white dotted line was the metastatic lesion. (C) The lung nodules and (D) The lung weight in each group on Day 26. LA content in (E) lung and (F) serum tissues. (G) Tumor lesions in the lung tissues stained with CRT, HMGB1, and TUNEL. (H) Immune cell infiltration in lung. (I‐L) IL‐6, IL‐10, TNF‐α and IL‐12 cytokines in lung tissue. Data represent mean ± SD (*n* = 3 or 5). Statistical comparisons were performed using one‐way ANOVA. *p < 0.05; **p < 0.01; ***p < 0.001.

Lung cancer model was firstly established by intravenously injecting LLC cells into C57BL/6N mice to evaluate the inhibition effect on primary tumor, and these mice was divided into four groups treated with PBS, DCA, PDIC‐AC, and PDIC‐NAC, respectively (Figure 6A). After 10‐day treatment, the number of pulmonary nodules in PDIC‐AC group was significantly reduced to 6, which is considerably lower than that in PBS (30), DCA (17) and PDIC‐NAC (20) groups (Figure 6B,C). Consistently, the lung weight in PDIC‐AC (150.6 mg) is also far less than that in PBS (362.9 mg), DCA (268.8 mg) and PDIC‐NAC (274.74 mg) (Figure 6D), confirming the most significant inhibition efficiency of PDIC‐AC on lung cancer. Additionally, no significant body weight loss was observed in mice treated with each compound, indicating that mice were well‐tolerated to all tested drugs at the therapeutic doses (Figure S45).

We further examined LA levels in serum and lung tissue following treatments. Consistent with in vitro detection results, PDIC‐AC group displayed a remarkable reduction of LA content by about 34% in serum and 50% in lung tissue, significantly exceeding the reduction amount observed in PDIC‐NAC group (20% in serum and 26% in lung tissue) (Figure 6E,F). Subsequently, we further conducted a comprehensive analysis of TAMs phenotypes by flow cytometer within lung tissue. Statistical results indicate that M1/M2 ratios in PBS, DCA, PDIC‐AC, and PDIC‐NAC groups were 0.51, 0.98, 5.80, and 0.97, respectively (Figure 6H and Figure S46), which underscores PDIC‐AC can efficiently orchestrate macrophage polarization from immunosuppressive M2 towards anti‐tumorigenic M1 phenotype. Furthermore, similar ICD activation was also found in PDIC‐AC treated tumor lesions in lungs as evidenced by the simultaneous upregulation of surface‐exposed CRT and the concurrent efflux of nuclear HMGB1 (Figure 6G). The release of DAMPs can recruit and activate DCs, which in turn stimulate T cells. We therefore examined the ratios of T cells in lungs, and statistical results indicate that the proportions of the helper T cell, cytotoxic T cells, and activated CD8^+^ T cells in PDIC‐AC group were 2.09, 1.68, and 1.36 times higher than that in PBS group, respectively, which providing further evidence for effective immune response to inhibit tumor growth (Figure 6H and Figures S47 and S48). Analysis of T‐lymphocyte typing subsets in spleen further indicates that PDIC‐AC group mounted the most potent immune response. These results conclusively demonstrated that PDIC‐AC treatment alleviates the acidic tumor microenvironment and activate ICD, thereby promoting immune cell infiltration and activation.

Consistent with the substantial immune cell infiltration, cytokine profiling of lung tissue via enzyme linked immunosorbent assay (ELISA) identified that PDIC‐AC can elevate the pro‐inflammatory factor of TNF‐α, IL‐6, and IL‐12 to 210.71%, 207.78%, and 300% of the PBS group, respectively, while reducing anti‐inflammatory factor IL‐10 by 50.02% (Figure 6I–L). This robust cytokine confirmed that PDIC‐AC effectively reverses the immunosuppressive tumor microenvironment, highlighting its significant immunotherapeutic potential. Assessment of apoptosis in tumor lesions by terminal deoxynucleotidyl transferase mediated dUTP nick‐end labeling (TUNEL) assay revealed that PDIC‐AC induced tumor cell death more effectively than either DCA or PDIC‐NAC (Figure 6G), as validated by a markedly higher percentage of TUNEL‐positive cells.

Collectively, these findings establish PDIC‐AC as a potent chemo‐immunotherapeutic agent that not only directly inhibits tumor cell metabolism and promotes apoptosis in tumor cells, but also reverses the immunosuppressive tumor microenvironment to elicit antitumor immunity.

### 2.9 PDIs Induce Distal Antitumor Effect on Subcutaneous Tumor

**FIGURE 7:**
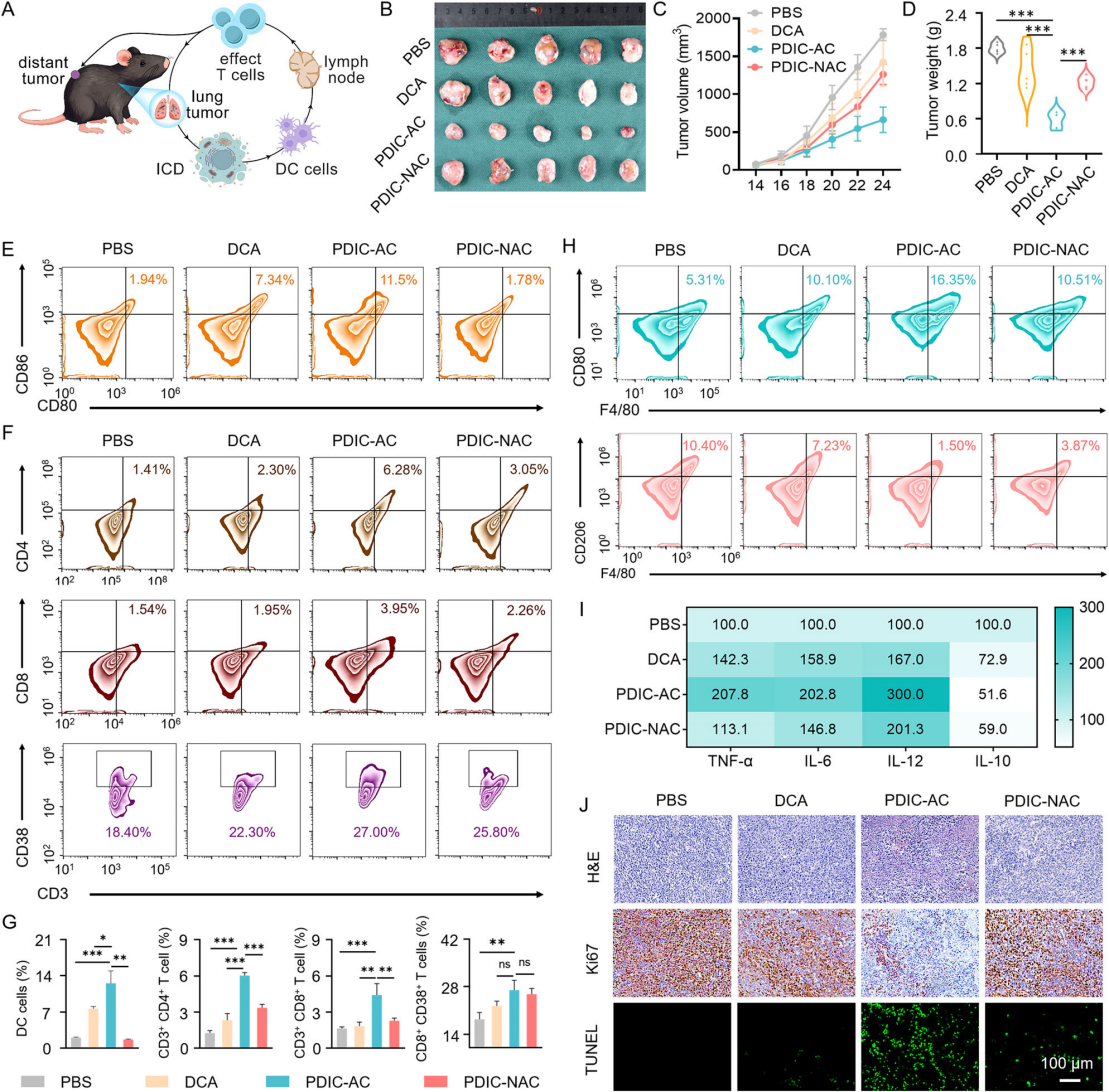
PDIs induce distal antitumor effect. (A) The immunological mechanism of local treatment‐induced distal effects. (B) Photographs of tumor tissues, (C) monitoring tumor volume and (D) tumor weight in the different treatment groups. (E) DC cells in lymph node. (F) The helper T cells, CD8^+^ T cells and activated CD8^+^ T cells in distal tumor. (G) Quantitative results of DC cells in lymph node, and the helper T cells, CD8^+^ T cells and activated CD8^+^ T cells in distal tumor. (H) Macrophage phenotyping in distal tumor. (I) IL‐6, IL‐10, TNF‐α and IL‐12 cytokines in tumor. (J) H&E, Ki‐67, and TUNEL of subcutaneous tumor. Data represent mean ± SD (*n* = 3 or 5). Statistical comparisons were performed using one‐way ANOVA. *p < 0.05; **p < 0.01; ***p < 0.001.

Extensive studies have shown that local treatments (e.g., chemotherapy intervention) can induce ICD in tumor cells, thereby releasing a substantial tumor‐specific antigens, which can successfully transform local inflammation into systemic immune activation, enabling immune cells to autonomously recognize and attack distant lesions (Figure 7A) [61, 62]. Therefore, we further conducted a statistical analysis of the immune activation in distal tumor.

Following 10 d of treatment, gross observation of dissected tumors at the endpoint visually showed that the PDIC‐AC group harbored the smallest tumor lesions, followed by the PDIC‐NAC group, whereas DCA only exhibited a weak inhibitory effect on tumor growth (Figure 7B). As revealed by the dynamic tumor growth curves and endpoint tumor weight measurements, a marked suppression of tumor volume in PDIC‐AC group (663 mm^3^) was observed, which significantly outperforming both DCA (1425 mm^3^) and PDIC‐NAC group (1263 mm^3^), thereby underscoring the exceptional anti‐tumor efficacy of PDIC‐AC (Figure 7C,D). However, as previously mentioned, PDIC‐AC shows minimal accumulation in subcutaneous tumor tissue. Therefore, this provides compelling evidence that PDIC‐AC not only treats existing tumor lesions within the lung but also activates systemic immunity through localized pulmonary therapy, thereby effectively inhibiting the growth of distant tumor tissues.

Flow cytometry analysis of distal tumors was performed to quantify tumor‐infiltrating immune cells, including DC cells, macrophages and T cells. As shown in Figure 7E, the proportion of DCs in PDIC‐AC group was 5.93, 1.57, and 6.46 folds higher than that in PBS, DCA, and PDIC‐NAC groups, respectively. We further assessed T cells activation in distant tumors. As anticipated, PDIC‐AC group showed an increase of 4.45 holds in helper T cell population, and an increase of 2.56 and 1.47 folds in cytotoxic T cells and activated CD8^+^ T cells, respectively, compared to the PBS group (Figure 7F,G). Concurrently, the ratio of M1/M2 macrophages in PDIC‐AC group was 13.85, 9.89 and 6.38 folds of that in PBS, DCA and PDIC‐NAC group, respectively (Figure 7H and Figure S49). The above results indicate that PDIC‐AC exhibits the most effective in reversing the immunosuppressive TME. We further measured the key cytokines levels in tumor associated with immune cells, including the pro‐inflammatory and the anti‐inflammatory factors. As shown in Figure 7I, PDIC‐AC group exhibited the most pronounced shift towards a pro‐inflammatory state, characterized by the highest levels of TNF‐α, IL‐6, and IL‐12, and the most significant reduction in IL‐10. This cytokine profile indicates that PDIC‐AC can enhance the host's immune response against the tumor. H&E staining of major organs revealed no damage following administration of DCA, PDIC‐AC, and PDIC‐NAC (Figure S50). Evaluation of blood routine and biochemistry markers, including hemoglobin (HGB), red blood cell distribution width (RDW), and aspartate transaminase (AST), etc., indicated that these parameters were within normal ranges after treatment with these compounds, underscoring the favorable biosafety profile of DCA, PDIC‐AC, and PDIC‐NAC at therapeutic doses (Figure S51). Overall, this work confirms that PDIC‐AC can effectively remodel the tumor‐suppressive microenvironment and stimulate potent distant antitumor immune responses, demonstrating significant potential for immunotherapy.

## 3 Conclusion

In summary, we herein grafted DCA into PDIs via ionic bonds and covalent bonds to develop PDIC‐AC and PDIC‐NAC, respectively. Research findings indicate that ionic bond effectively remodels the surface electrostatic potential and cell distribution of PDIC‐AC, which collectively enhances the ability of PDIC‐AC to inhibit PDHK1 and generate ROS, thereby achieving potent energy depletion to initiate cell apoptosis. Moreover, PDIC‐AC reduces the secretion of LA to relieve the immunosuppression by HIF‐1α signal pathway and promote the polarization of macrophages from a pro‐tumor M2 phenotype towards an anti‐tumor M1 phenotype. In addition, excessive ROS activate PERK‐eIF2α‐ATF4‐CHOP oxidative stress pathway to initiate ICD effect and immune response. In vivo antitumor studies confirmed that PDIC‐AC not only exhibits excellent chemo‐immunotherapy efficacy in primary lung cancer, but also inhibits the distant tumor growth by activating systemic immune response. Collectively, this work establishes PDIC‐AC as an energy metabolism‐targeting immunotherapeutic agent characterized by energy depletion, alleviation of the immunosuppressive microenvironment and activation of adaptive immunity. Simultaneously, this work provides mechanistic insights for chemical bond engineering‐modulated biological activity.

## 4 Experimental Section

### 4.1 Statistical Analysis

The experimental results were analyzed using GraphPad Prism 8.0 software. One‐way analysis of variance (ANOVA) was performed, followed by Tukey's post hoc test for multiple comparisons. A *p* value of less than 0.05 was considered statistically significant. Significant differences between groups are indicated in the figures as *P<0.05, **P<0.01, and ***P < 0.001.

## Author Contributions

**Xuejie Zhao**: conceptualization, methodology, investigation, data curation, visualization, and Writing – original draft; **Yuting Liu**: conceptualization, investigation, data curation, and visualization. **Xiao Huang**: investigation and formal analysis. **Liwen Zhao**: investigation. **Chunli Li**: writing – supervision, methodology, and review and editing. **Yongwei Huang**: funding acquisition, writing – review and editing, supervision, and project administration.

## Ethics Statement

All animal experiments have been approved by the Animal Management and Ethics Committee of Henan University (Nos: HUSOM2024‐005). Female C57BL/6N mice (18‐20 g) were obtained from Beijing Charles River and housed under specific pathogen‐free conditions. In accordance with the *Guide for the Care and Use of Laboratory Animals* and NIH Guidelines for Humane Endpoints in Tumor Studies, Mice will be humanely euthanized immediately if any of the following criteria are met: tumor volume over 2000 mm^3^, body weight loss greater than 20% of initial weight, or severe distress signs including lethargy and hunched posture.

## Conflicts of Interest

The authors declare no conflicts of interest.

## Supporting information




**Supporting File**: advs77135‐sup‐0001‐SuppMat.docx.

## Data Availability

The data that support the findings of this study are available from the corresponding author upon reasonable request.
