# Supplementary material for: Chemical Bond‐Orchestrated Energy Depletion to Augment Lung Cancer Chemo‐Immunotherapy
Source: Adv Sci (Weinh). 2026 Aug 11:e77135. Online ahead of print. doi: 10.1002/advs.77135 (PMC13460281; doi:10.1002/advs.77135)
Supplement: Supplementary file 1 [file ADVS-9999-e77135-s001.docx]

Supporting information for

**Chemical Bond-Orchestrated Energy Depletion to Augment Lung Cancer Chemo-Immunotherapy**

Xuejie Zhao, Yuting Liu, Xiao Huang, Liwen Zhao, Chunli Li* and Yongwei Huang*

Laboratory for NanoMedical Photonics, School of Basic Medical Science, Henan University, Kaifeng 475004, China.

*Corresponding author: Chunli Li ([licl@henu.edu.cn](mailto:licl@henu.edu.cn)); Yongwei Huang (huangyw@henu.edu.cn).

Table of contents

[Table of contents	2](#_Toc235141832)

[Experimental Procedures	4](#_Toc235141833)

[**Table S1.** Primers used for qPCR.	15](#_Toc235141834)

[**Table S2.** Relative LA content in WT and KD LLC cell and in supernatant.	15](#_Toc235141835)

[**Table S3.** Relative fluorescence intensity of HIF-1α and ARG1 protein in RAW 264.7 cells.	16](#_Toc235141836)

[**Table S4.** Relative ratio of M1/M2 phenotype.	16](#_Toc235141837)

[**Table S5.** Relative content of cytokines in supernatant of WT and KD LLC cells.	16](#_Toc235141838)

[**Table S6.** In vivo pharmacokinetic parameters of PDIC-AC in blood.	17](#_Toc235141839)

[**Figure S1.** Synthesis process of PDIC-AC and PDIC-NAC.	17](#_Toc235141840)

[**Figure S2.** The ^1^H NMR spectrum of PDIC-AC.	17](#_Toc235141841)

[**Figure S3.** The ^13^C NMR spectrum of PDIC-AC.	18](#_Toc235141842)

[**Figure S4.** High resolution ESI-MS of PDIC-AC.	19](#_Toc235141843)

[**Figure S5.** The ^1^H NMR spectrum of PDIC-NAC.	20](#_Toc235141844)

[**Figure S6.** The ^13^C NMR spectrum of PDIC-NAC.	20](#_Toc235141845)

[**Figure S7.** High-resolution ESI-MS of PDIC-NAC.	21](#_Toc235141846)

[**Figure S8.** Optimized electrostatic potential of PDIC-NAC (left) and PDIC-AC^+^ (right).	22](#_Toc235141847)

[**Figure S9.** The pKa, UV-Vis spectra and the size distribution of PDIC-ACin PBS +10%FBS.	22](#_Toc235141848)

[**Figure S10.** Statistics on PDHK1 gene expression in LUAD and LUSC	23](#_Toc235141849)

[**Figure S11.** Cell viability of LLC after different treatments.	23](#_Toc235141850)

[**Figure S12.** Surface electrostatic potential distribution of PDHK1 Kinase.	23](#_Toc235141851)

[**Figure S14.** Analysis of the pcDNA3.1-C-3×Flag Vector Map.	25](#_Toc235141852)

[**Figure S15.** The partial sequence alignment of wild type PDHK1 and mutant PDHK1.	25](#_Toc235141853)

[**Figure S16.** The quantification results of the Flag tagged mutant PDHK1 and Flag tagged wild type PDHK1 thermal stability in CETSA.	25](#_Toc235141854)

[**Figure S17.** Results of the EC_50_ value of (A) DCA, (B) PDIC-AC, and (C) PDIC-NAC on PDHK1 enzyme activity..	26](#_Toc235141855)

[**Figure S18.** Western blot and qPCR validation of PDHK1 expression levels in LLC cells.	26](#_Toc235141856)

[**Figure S19.** Immunofluorescence detection of HIF-1α in RAW 264.7 after treatment by conditioned medium collected from LLC cells.	26](#_Toc235141857)

[**Figure S20.** Immunofluorescence detection of ARG1 in RAW 264.7.	27](#_Toc235141858)

[**Figure S21.** Flow cytometry detection of M1 or M2 macrophage phenotyping of RAW 264.7 after different treatments and quantification results.	27](#_Toc235141859)

[**Figure S22.** Detection of cytokines in conditioned medium after cultured RAW 264.7 cells collected from PDIC-AC treated KD-LLC cells..	27](#_Toc235141860)

[**Figure S24.** Immunofluorescence detection and quantitative results of HIF-1α in RAW 264.7 cells..	28](#_Toc235141861)

[**Figure S25.** Immunofluorescence detection and quantitative results of ARG1 in RAW 264.7..	29](#_Toc235141862)

[**Figure S27.** Macrophage typing detection quantitative results..	29](#_Toc235141863)

[**Figure S28.** The levels of IL-6, IL-12, TNF-α, and IL-10 secreted by the macrophages after incubating with the supernatants of WT-LLC cells..	30](#_Toc235141864)

[**Figure S29.** The fluorescence quantification of total ROS in LLC cells after different treatments..	30](#_Toc235141865)

[**Figure S30.** Flow cytometry analysis and fluorescence quantification of total ROS in LLC cells ..	30](#_Toc235141866)

[**Figure S31.** Representative fluorescence images and quantification of (A) H_2_O_2_, (B) O_2_^•-^, (C) ^•^OH in LLC cells after different treatments..	31](#_Toc235141867)

[**Figure S32.** Flow cytometry analysis and quantification of (A) O_2_^•-^, (B) H_2_O_2_, (C) ^•^OH in LLC cells after different treatment..	32](#_Toc235141868)

[**Figure S33.** Relative expression of (A) D-LOOP, (B) Cyt b, (C) ND4, (D) Cox III and (E) ATP synthase subunits in PBS, DCA, PDIC-AC and PDIC-NAC treatment groups.	32](#_Toc235141869)

[**Figure S34.** Quantitative analysis of JC-1 aggregates/JC-1 monomers of LLC cells.	33](#_Toc235141870)

[**Figure S35.** Basal Respiration, Maximal Respiration, ATP Production, and Spare respiratory capacity after different treatments.	33](#_Toc235141871)

[**Figure S36.** Quantitative results of LLC cell examined by flow cytometry. .	33](#_Toc235141872)

[**Figure S37.** Flow cytometric analysis of apoptosis ratios in LLC cells following treatment with PDIC‑AC combined with exogenous ATP or methyl pyruvate and quantitative results. .	34](#_Toc235141873)

[**Figure S38.** Western blot detection of apoptosis-related proteins.	34](#_Toc235141874)

[**Figure S40.** Flow cytometry detection of the DC cells and quantitative results..	35](#_Toc235141875)

[**Figure S41.** Flow cytometry detection of T cells and activated CD8^+^ T cells in spleen and in tumors.	36](#_Toc235141876)

[**Figure S42.** Biosafety evaluation of PDIC-AC and PDIC-NAC..	36](#_Toc235141877)

[**Figure S43.** H&E staining images of main organs from mice treated with PDIC-AC	37](#_Toc235141878)

[**Figure S44.** Fluorescence images of main organs in vitro collected from mice after intravenously injected PDIC-AC at the dose of 2 mg kg^-1^ body weight.	37](#_Toc235141879)

[**Figure S46.** Quantitative results of macrophage phenotyping in lung tissue..	38](#_Toc235141880)

[**Figure S47.** Flow cytometry analysis and quantification of the proportion of T cells in mice bearing primary lung tumors.	38](#_Toc235141881)

[**Figure S48.** Flow cytometry analysis and quantification of the proportion of T cells in spleens.	39](#_Toc235141882)

[**Figure S49.** Flow cytometry detection of macrophages in distant tumors and quantitative results..	39](#_Toc235141883)

[**Figure S50.** H&E images of the heart, liver, spleen, and kidneys of different treatment groups.	40](#_Toc235141884)

[**Figure S51.** Blood routine and blood biochemical index of the mice treated with different treatments.	40](#_Toc235141885)

Experimental Procedures

**Materials.**

All starting materials and reagents were commercially available. All biological reagents purchased from commercial suppliers including Beyotime Biotechnology, Corning Biotechnology, Vazyme Biotechnology, MEC, Proteintech Biotechnology Co. Ltd. (Wuhan, China), Elabscience Biotechnology Co. Ltd. (Wuhan, China), Zenbio Biotech (Sichuan, China), Shanghai Enzyme-Linked Biotechnology Co., LTD (Shanghai, China), Sangon Biotech (Shanghai, China). Solarbio Science & Technology Co., Ltd. (Beijing, China), etc.

**Characterization.**

^1^H NMR (400 MHz, 500 MHz) and ^13^C NMR (125 MHz) spectra were recorded on Bruker spectrometer. Chemical shifts in ^1^H NMR spectra were recorded in delta (*δ*) units, parts per million (ppm) relative to DMSO-*d*_6_ (*δ* = 2.50 ppm). Chemical shifts in ^13^C NMR spectra were recorded in delta (*δ*) units, parts per million (ppm) relative to CF_3_COOD (*δ* = 164.2, 116.6 ppm) as solvent. Mass spectra were recorded on a Thermo Scientific Q Exactive HF Orbitrap-FTMS (Thermo Fisher Scientific, United States). Absorption and emission spectra were recorded on UV-3600Plus spectrophotometer (Shimadzu Corporation, Japan) and Spectrofluorometer FS5 (Edinburgh Instruments, United Kingdom) respectively. Confocal microscopy was performed on a LEICA TCS SP8+STED confocal microscope (Leica Microsystems, Germany). Flow cytometry data was acquired using a cytoflex flow cytometer (BECKMAN COULTER Life Sciences, USA) and analyzed with Flowjo software. The in vivo imaging was carried out using an IVIS Lumina XRMS Series III in vivo imaging system (PerkinElmer, United States).

**Structural characterization of PDIC-AC and PDIC-NAC. PDIC-AC:** ^1^H NMR (400 MHz, DMSO-*d*_6_) *δ* 8.62 (s, 4H), 8.02 (s, 6H), 5.69 (s, 2H), 4.37 (s, 4H), 3.20 (t, *J* = 6.0 Hz, 4H); ^13^C NMR (125 MHz, CF_3_COOD) *δ* 173.0, 167.5, 138.8, 136.4, 133.3, 132.5, 125.4, 123.8, 65.0, 42.8, 40.7. HRMS (ESI-FTMS) m/z: [M]^2+^ Calcd for C_28_H_18_Cl_4_N_4_O_4_, 307.0036. found: 307.0033. **PDIC-NAC:** ^1^H NMR (500 MHz, DMSO-*d*_6_) δ 8.75

(t, *J* = 6.0 Hz, 2H), 8.58 (s, 4H), 6.37 (s, 2H), 4.25 (t, *J* = 5.5 Hz, 4H), 3.58-3.50 (m, 4H); ^13^C NMR (125 MHz, CF_3_COOD) δ 171.1, 167.3, 138.7, 136.4, 133.2, 132.3, 125.4, 124.0, 66.9, 42.1, 41.1. HRMS (ESI-FTMS) m/z: [M+H]^+^ Calcd for C_32_H_17_O_6_N_4_Cl_8_, 832.8651; found: 832.8645.

**UV-Vis and fluorescence spectra.** UV-Vis spectra: 60 μL (2 mM) of PDIC-AC and PDIC-NAC were added to 3 mL H_2_O to give a final concentration of 40 μM, and the spectrum were recorded using a UV-Vis spectrometer. Fluorescence spectra: 5 μL (2 mM) of PDIC-AC and PDIC-NAC were diluted with 3 mL H_2_O to give a final concentration of about 3.33 μM, and the spectrum were recorded using a fluorescence spectrometer.

**Calculation of pKa.** PBS buffers at pH values of 3, 3.5, 4, 4.5, 5, 5.5, 6, 6.5, 7, 7.5, 8, 8.5, 9, 9.5, 10, 10.5, and 11 were prepared. To each buffer, 40 μL of PDIC-AC (2 mg/mL) was added, and the mixtures were incubated for 3 h. After incubation, 350 μL of the supernatant from each sample was transferred to a 96‑well plate, and the absorbance at 524 nm was measured using a microplate reader. The minimum absorbance (A_min_) and maximum absorbance (A_max_) were determined. A scatter plot was generated with pH as the abscissa and log [(A − A_min_)/(A_max_ − A)] as the ordinate. The pKa value was determined as the x‑intercept of the fitted curve.

**ROS detection.** Cells in the logarithmic growth phase, cultured in 2 mm dishes, were treated with DCA, PDIC-AC, and PDIC-NAC (2 μM) for 8 h. The working solution of 2′,7′-dichlorodihydrofluorescein diacetate (DCFH-DA) was added to the cells (500 μL per well) and incubated for 30 min. Afterward, the medium was removed, and the cells were washed with PBS to remove excess dye, followed by the addition of 1 mL serum-free medium prior to imaging using confocal laser scanning microscopy (CLSM). Similarly, dihydroethidium (DHE, a superoxide anion probe), peroxyfluor-1 (a hydrogen peroxide probe), hydroxyphenyl fluorescein (HPF, a hydroxyl radical probe), and singlet oxygen sensor green (SOSG, a singlet oxygen probe) were used to detect different reactive oxygen species (ROS). Additionally, ROS levels were further assessed using flow cytometry. Briefly, logarithmic-phase LLC cells were treated with DCA (2 mM), PDIC-AC (2 μM), or PDIC-NAC (2 μM) for 8 h, then harvested by centrifugation and stained with DCFH-DA, DHE, peroxyfluor-1 and HPF in the dark at 37 °C for 30 min. Stained cells were analyzed using CytoFLEX flow cytometer, and data were processed with FlowJo software.

**Optimized electrostatic potential of PDIC-NAC and PDIC-AC.** The molecular structure models were constructed using GaussView 5.0.9 software. The ground-state geometry was fully optimized using density functional theory (DFT) with the B3LYP functional and the 6-31G (d) basis set, and set the charge of PDIC-NAC to 0 and the charge of PDIC-AC to +2. All calculations were carried out using the Gaussian 09 program package.

**Determination of the reduction potential.** In this experiment, the reduction potentials of two compounds, PDIC-AC and PDIC-NAC, were determined using cyclic voltammetry. Each compound was first dissolved in dimethyl sulfoxide (DMSO) to a concentration of 0.2 mM, followed by the addition of tetrabutylammonium hexafluorophosphate (TBAPF₆) as the supporting electrolyte to a final concentration of 0.1 M. Electrochemical measurements were performed using a three-electrode system consisting of a glassy carbon working electrode, an Ag/AgCl reference electrode, and a platinum wire counter electrode. The scan rate was 20 mV/s, and the potential range was from -2 V to 0 V.

**In vitro reduction assay of PDIC-AC and PDIC-NAC.** The reaction mixture consisted of 0.5 mg/mL mouse liver microsomes as the enzyme source, 1 mM NADPH as the electron donor, and 40 μM PDIC-NAC or PDIC-AC as the electron acceptor in 0.1 M phosphate buffer (PBS). The mixture was incubated in the dark at 37 °C for 24 h with gentle shaking, followed by centrifugation at 12,000×g for 10 min at 4 °C to precipitate proteins. Control samples without PDIC-AC or PDIC-NAC.

**Verification of mtDNA Damage by qPCR.** LLC cells were cultured overnight in cell culture dishes and then incubated with various treatments: DCA (2 mM), PDIC-AC (2 μM), or PDIC-NAC (2 μM) for 8 h. After incubation, the cells were washed with PBS and harvested by centrifugation. Total RNA was extracted using an RNA extraction kit, and reverse transcription was performed. Quantitative real‑time PCR (qPCR) was carried out using primers targeting displacement loop region (D-LOOP), Cyt b, ATP6, ND4, and Cox III. GAPDH was used as an internal control, and relative gene expression was calculated using the 2^ΔΔCt method.

**Pull-down experiment.** A549 cells were lysed in immunoprecipitation (IP) lysis buffer (50 mM Tris-HCl, pH 7.4, 150 mM NaCl, 0.5% NP-40, 1 mM EDTA, 10% glycerol) supplemented with protease and phosphatase inhibitors. After incubation on ice for 30 min, the lysates were centrifuged at 12,000×g for 10 min at 4 °C. Protein concentrations were determined using the BCA assay and adjusted to 2~3 mg/mL. The protein supernatant was evenly divided into two aliquots, which were incubated with 50 μM biotin (negative control) or 50 μM Bio-PDIC-BH (test group) for 4 h at 4 °C with gentle rotation. Subsequently, the streptavidin magnetic beads were washed with IP lysis buffer, and the compound–protein–bead complexes were collected for Western blot (WB) analysis.

**Competitive pull-down assay.** To evaluate the specific binding interaction between PDIC-BH and its target protein, a competitive binding assay was performed using biotin-labeled PDIC-BH (Bio-PDIC-BH). Cell lysates were incubated with increasing concentrations of unlabeled PDIC-BH (25, 50, and 100 μM) at 4 °C for 4 h to compete for protein binding sites. Bio-PDIC-BH was then added at a fixed concentration, and the mixtures were incubated at 4 °C for 4 h with gentle rotation. Streptavidin-coated magnetic beads were added to each sample and incubated at 4 °C for 4 h to capture biotinylated complexes. The beads were washed three times with ice-cold lysis buffer to remove non‑specifically bound proteins. Bound proteins were eluted by boiling the beads in SDS‑PAGE loading buffer and analyzed by Western blot using specific primary antibodies. Band intensities were quantified to assess the dose‑dependent inhibition of Bio-PDIC-BH binding by free PDIC-BH, confirming the specificity of the interaction.

**Cellular thermal shift assay (CETSA).** After lysis using cell lysis buffer for WB and immunoprecipitation (containing 20 mM Tris, 150 mM NaCl, 1% Triton X-100, sodium pyrophosphate, β-glycerophosphate, EDTA, Na₃VO₄, and leupeptin), the soluble protein fraction was isolated by centrifugation at 12,000 rpm for 15 min at 4 °C. The lysate of LLC cells was treated with different compounds or vehicle, harvested, and aliquoted. The aliquots were subjected to a temperature gradient (42.5, 45, 47.5, 50, 52.5, 55, 57.5, 60, 62.5, and 65 °C) for 3 min in a thermal cycler, followed by centrifugation at 12,000 rpm for 15 min at 4 °C.

**Molecular docking simulation.** Molecular docking simulation was performed to predict the binding mode and affinity of PDIC-AC and PDIC-NAC toward PDHK1 kinase. The crystal structure of PDHK1 (PDB ID: 2Q8G) was retrieved from the RCSB Protein Data Bank. All water molecules and the native ligand were removed. Hydrogen atoms and Kollman charges were added using AutoDock Tools 1.5.6. The three-dimensional structures of DCA, PDIC-AC, and PDIC-NAC were energy-minimized using the MM2 force field in Chem3D and converted to PDBQT format with Open Babel. The grid box was centered on the active site (center: X= 1.507, Y= 39.897, Z= -9.399) with a size of 40×40×40 Å³ for DCA binding to pyruvate site, was centered on the active site (center: X= -3.463, Y= 42.361, Z=8.897) with a size of 36×34×68 Å³ for PDIC-AC binding to lipoamide site, and was centered on the active site (center: X= -2.666, Y= 42.679, Z=4.125) with a size of 40×40×78 Å³ for PDIC-NAC binding to lipoamide site. Docking simulations were carried out using AutoDock Vina 1.1.2 with an exhaustiveness value of 32. The best pose for each ligand, based on the lowest binding energy, was selected for further analysis of protein-ligand interactions using PyMOL 2.5.

**Enzyme activity assay.** The PDHK1 enzyme, kinase assay buffer and PDHA1 substrate were thawed on ice. A pre-cooled 96-well plate was used to prepare the reaction system. The enzyme was diluted to the desired concentration in kinase assay buffer and 3 μL of the diluted solution was transferred to a 384-well plate. Various concentrations of DCA, PDIC-AC and PDIC-NAC were then added and pre-incubated for the indicated duration. Then, 2 μL of the substrate/ATP mixture was added to initiate the reaction and was allowed to proceed for 40 min at room temperature. The reaction was terminated by adding 5 μL of ADP-Glo™ reagent to each well, followed by an incubation period of 40 minutes at room temperature. Next, 10 μL of kinase detection reagent was added to each well, after which the plate was incubated for 30 min at room temperature. Luminescence was then measured using an ELISA microplate reader.

**Cell culture.** Mouse-derived lewis lung cancer cells (LLC), human-derived non-small cell lung cancer cell A549, and mouse-derived alveolar epithelial cells (MLE-12) were cultured in an incubator using high-sugar DMEM medium (containing 10% fetal bovine serum, 100 U mL^-1^ penicillin, and 0.1 mg mL^-1^ streptomycin).

**Cell viability assay.** Cells were seeded into 96-well plates at a density of 5,000 cells per well for proliferation assessment. After incubation under controlled conditions, the cells were treated with various concentrations of the test compounds upon reaching approximately 70% confluence. Subsequently, the wells were rinsed with PBS and incubated with 3-(4,5-dimethylthiazol-2-yl)-2,5-diphenyltetrazolium bromide (MTT) solution at 37 °C for 4 h. The MTT solution was then removed, and 100 μL of dimethyl sulfoxide (DMSO) was added to each well to dissolve the formazan crystals. Absorbance was measured at 570 nm after complete dissolution, and the half-maximal inhibitory concentration (IC₅₀) was determined using Prism 8.0.2 software.

**Co-localization imaging experiments.** LLC cells were cultured in 20 mm confocal dishes at 37 °C with 5% CO₂ for 24 h. The medium was then replaced with fresh medium containing 2 mM DCA, 2 μM PDIC-AC, or 2 μM PDIC-NAC, and the cells were incubated for 4 h. After washing three times with PBS, the cells were incubated with mitochondrial green fluorescent probe working solution (MitoTracker probe: serum-free medium = 1:1000) at 37 °C for 30 min. Following another wash with PBS, serum-free medium was added, and compound localization was observed using confocal laser scanning microscopy (CLSM).

**Lactate (LA) content test.** LLC cells were seeded in a 6-well plate and incubated at 37 ^o^C for 24 h. The cells were then treated with DCA (2 mM), PDIC-AC (2 μM), and PDIC-NAC (2 μM) for 12 h. After treatment, the medium was replaced with 2 mL of fresh medium, and the cells were cultured for an additional 12 h. The culture supernatant was collected and stored at 4 °C for subsequent LA content analysis. Subsequently, the cells (approximately 1×10^6^) were then washed and resuspended in 300 μL PBS (0.01 M, pH 7.4). Cell lysis was performed using a ultrasonicator, and the lysate was centrifuged. The resulting supernatant was used for protein concentration determination and further analysis via an ELISA kit, following the manufacturer's instructions.

**LA rescue experiment.** Conditioned medium from PDIC-AC treated cells was collected, and exogenous LA was added to achieve final concentrations consistent with the PBS control group. The resulting medium was then used to treat macrophages. Conditioned medium without LA supplementation served as the control, and a solvent control group was also included to exclude potential interference from the vehicle. By comparing functional parameters of macrophages (e.g., cytokine secretion, expression of polarization markers) among the groups, the reversal effect of exogenous LA on the immunomodulatory function of PDIC-AC was evaluated.

**Transient transfection experiment.** LLC cells were seeded in 6-well plates and cultured until reaching approximately 50% confluence. The culture medium was then replaced with 2 mL of fresh medium. A siRNA premix was prepared by combining 42.5 μL of Buffer, 75 pmol of siRNA, and 7.5 μL of transfection reagent. After mixing, the premix was added to the culture dishes. Following 48 h of incubation, total RNA was extracted to analyze PDHK1 mRNA expression levels. After 60 h of incubation, protein was extracted to evaluate PDHK1 protein expression levels.

**Plasmid construction and transfection.** The coding sequences of wild-type PDHK1 and mutant PDHK1 (Q61A/S75A) were synthesized by Genomeditech, and a Flag tag was introduced at the C-terminus. The constructed plasmids were verified by DNA sequencing prior to use in subsequent cell transfection experiments. LLC cells were seeded into culture dishes and cultured until reaching 70%–80% confluence. The Flag tagged wild-type and mutant PDHK1 plasmids were then transfected into the cells using Lipofectamine 3000 according to the manufacturer’s instructions. After 48 h of transfection, the cells were harvested for Western blot analysis to confirm the expression of exogenous PDHK1 proteins.

**The detection of extracellular acidification rate (ECAR).** Cells were seeded into Seahorse-specific microplates and cultured to an appropriate density, followed by pretreatment with DCA, PDIC-AC, or PDIC-NAC. The medium was then replaced with fresh basal medium. According to the manufacturer’s instructions, glucose, oligomycin, and 2-deoxyglucose were sequentially injected according to the preset dosing protocol, and real-time pH changes in each well were monitored using microsensors.

**The detection of oxygen consumption rate (OCR).** The steps are as follows: 1. Cell culture and plate spreading: Inoculate the LLC cells to be tested into dedicated Seahorse microplates at an optimized density and culture them in 37℃, 5% CO_2_ until a suitable monolayer is formed. 2. Reagent preparation. Prepare the detection medium and redissolve DCA, PDIC-AC and PDIC-NAC to be tested in the detection medium to the required concentration. 3. Hydration: Hydrate the Seahorse test kit overnight in a CO₂ -free incubator. 4. Load the prepared cell plates, hydrated test kits and drugs into the Seahorse XF analyzer, and the instrument will operate automatically. After the detection is completed, the software will export the OCR data at each time point.

**The detection of electron paramagnetic resonance (EPR).** LLC cells were cultured under standard conditions (37 °C, 5% CO₂) until reaching the required density (typically 80–90% confluence), and then treated with PDIC-AC, or PDIC-NAC. Subsequently, the cells were harvested by trypsinization, collected, and resuspended for EPR analysis.

**Mitochondrial network.** LLC cells were inoculated into laser confocal microdishes and grown for 24 h. Then, PDIC-AC (2 μM), PDIC-NAC (2 μM) and DCA (2 mM) were added and incubated for 12 h. Subsequently, the Mito-Tracker Red CMXRos working solution prepared in serum-free medium was changed and incubated in the dark for 25 min followed by washing off the excess dye with PBS for three times, and photographed with CLSM. The mitochondrial morphometric quantitative images acquired by Image J software.

**Mitochondrial membrane potential detection.** LLC cells in logarithmic growth phase were treated with DCA (2 mM), PDIC-AC (2 μM), PDIC-NAC (2 μM) for 8 h. Replace JC-1 working solution and incubate at 37 ºC for 20 min, then, washed twice with 1×JC-1 staining buffer. Add 1 mL of complete medium to be photographed under a confocal microscope.

**NAD+/NADH detection.** NADH/NAD+ in the cell were measured by NAD+/NADH detection kit with WST-8 method according to the protocols.

**Detection of ATP content.** Well-cultured LLC cells were seeded into a 6-well plate and then treated with DCA (2 mM), PDIC-AC (2 μM), or PDIC-NAC (2 μM) for 12 h. After treatment, the cells were lysed with cell lysis buffer, and the resulting supernatant was collected for further analysis. Following brief equilibration of the black 96-well plate on ice, the samples and detection solution were added, incubated under the specified conditions, and the signal was measured using an enzyme-linked assay.

**Apoptosis detection.** Once the desired density of LLC cells was reached in the 6-well plates, DCA, PDIC-AC and PDIC-NAC were introduced at doses of 2 mM, 2 μM and 2 μM respectively, and the cells were incubated for 24 h. The cells were then collected and resuspended in a binding buffer. The staining process involved adding 10 μL of 7-AAD and 5 μL of V-APC, followed by an incubation period of 5 min. Detection was then carried out using flow cytometry.

**Western blot.** LLC cells were seeded into 10 cm cell culture dishes and incubated until reaching approximately 70% confluence. The cells were then treated with DCA (2 mM), PDIC-AC (2 μM), or PDIC-NAC (2 μM). Subsequently, the cells were lysed on ice for 30 min using RIPA lysis buffer supplemented with PMSF and phosphatase inhibitors. The resulting supernatant was quantified using the BCA Protein Assay Kit. For Western blot analysis, protein samples were mixed with loading buffer and boiled for 15 min. The samples and molecular weight markers were then separated by electrophoresis on 12% SDS-PAGE gels at 80 V. Following electrophoresis, proteins were transferred onto membranes, with current intensity and transfer time adjusted as necessary. The target protein bands were identified based on the molecular weight markers, and the membranes were blocked with 5% skim milk or 5% BSA for 2 h. After blocking, the membranes were incubated with primary antibodies against the target proteins at 4 °C for 12 h, washed with TBST, and then incubated with the corresponding secondary antibodies at room temperature for 2 h. Finally, ECL ultrasensitive chemiluminescent substrate was applied to the membranes, and protein expression was visualized using a chemiluminescence imaging system.

**Cell cycle analysis.** The cells were cultured in 6-well plates and subjected to each treatment. The cell culture medium was supplemented with DCA (2 mM), PDIC-AC (2 μM) and PDIC-NAC (2 μM), after which the cells were incubated for 24 h. Then, the cells were harvested and washed with PBS, followed by adding 70% alcohol to the cells and incubated on ice for 24 h, the cells were washed with PBS and resuspended with 400 µL propidium cell cycle reagent. After incubation at 37 °C for 15 min, the cells were examined through CytoFLEX flow cytometer and analyzed with Flow J software.

**Macrophage induction.** LLC cells were seeded into culture plates and incubated at 37 °C until reaching 60% confluence. The medium was then removed and replaced with fresh medium. The cells were treated with DCA (2 mM), PDIC-AC (2 μM), or PDIC-NAC (2 μM) for 8 h, after which the medium was replaced with fresh medium and incubation continued for an additional 12 h. The resulting supernatant was collected and transferred to culture plates for subsequent culture of RAW264.7 cells.

**Immunofluorescence.** The cells were fixed with 4% paraformaldehyde for 10 min before being infiltrated with 0.5% Triton-X 100 for 5 min to allow the proteins to reach the nucleus, and then rinsed with PBS, sealed with 1% BSA for 1 h. Cells were treated with the primary antibody at 4 ^o^C, washed three times with PBS, and then incubated with the secondary antibody for 2 h at room temperature. The nuclei were stained for 3 min with a DAPI staining solution, then imaged using an inverted fluorescence microscope after adding an anti-fluorescence quencher.

**In vivo distribution.** C57BL/6N mice were injected with 100 μL (0.4 mg/mL) of PDIC-AC solutions via the tail vein, heart, liver, spleen, lung, and kidney tissues were removed after 6 h injection, and the tissues were photographed with small animal in vivo imaging system.

**Evaluation of ICD vaccine efficacy.** LLC cells were co‑incubated with 1, 2, or 4 μM PDIC-AC. The harvested cells were then subcutaneously injected into the right dorsal flank of mice. Seven days post‑vaccination, live LLC cells were subcutaneously injected into the left contralateral dorsal flank for tumor rechallenge. On day 14 after rechallenge, the mice were euthanized, and tumor tissues were excised. Tumor volume and weight were measured and statistically analyzed.

**Lung cancer model.** LLC cells in the logarithmic growth phase were resuspended in PBS at a concentration of 6×10^6^ cells/mL. These cells were then injected intravenously at a volume of 100 μL to establish a primary pulmonary tumor model. 14 days after the initial treatment, the animals were randomly assigned to four groups (n = 5 per group). The control group received PBS injections, while the DCA, PDIC-AC, and PDIC-NAC groups were treated with 2 mg·kg⁻¹ of the respective compounds via injection for 12 consecutive days. After completing the treatment regimen, the animals in the model group were humanely euthanised and their lungs carefully dissected for subsequent analysis.

**Distant tumor model.** LLC cells in the logarithmic growth phase were resuspended in PBS to a concentration of 6×10^6^ cells/mL before subcutaneously injecting 100 μL to establish the xenografted tumor model. Once the tumor volume reached approximately 100 mm³, the model animals were randomly divided into four groups of five mice each. The PBS group received an injection of saline, while the DCA, PDIC-AC, and PDIC-NAC groups were injected with 2 mg kg^-1^ of their respective substances over a 10-day treatment period. Following the treatment period, the model animals were euthanized, and the tumors were excised, weighed, photographed, and documented for further analysis.

**Detection of the percentage of immune cells in the tumor.** First, each tumor tissue sample was placed in one well of a 6-well plate. Then, 2 mL of DMEM medium containing collagenase IV and DNase I was added to each well. The tumor tissues were then cut into pieces measuring approximately 1 mm³ and digested at 37 °C for 45 minutes. Following digestion, the tissue pieces were sieved through a 100 µm sieve and the process was stopped by adding a termination solution (DMEM containing 2 mM EDTA). The sieved material was then centrifuged for five minutes at 1,500 rpm at room temperature and the resulting supernatant was discarded. The resulting precipitate was resuspended in PBS to remove connective tissue. This was followed by another round of centrifugation and discarding of the supernatant. The resultant precipitate comprised the cells intended for detection. M1 macrophages (PE anti-mouse F4/80, FITC anti-mouse CD11b, APC anti-mouse CD80); M2 macrophages (PE anti-mouse F4/80, FITC anti-mouse CD11b, APC anti-mouse CD206); CD4^+^ T lymphocytes (FITC anti-mouse CD3, APC anti-mouse CD4); CD8^+^ T lymphocytes (FITC anti-mouse CD3, APC anti-mouse CD8); activated CD8^+^ T lymphocytes (FITC anti-mouse CD3, APC anti-mouse CD8, PE anti-mouse CD38). The dye was added, followed by a 20-minute incubation, and then detected using flow cytometer.

**Detection of the percentage of immune cells in the lung.** Fresh lung tissues were placed in cold PBS, trachea and connective tissue were removed, an appropriate amount of digestive solution was added, and the tissue was cut into small pieces of about 1 mm^3^ in volume and incubated on a shaking table for 60 min. The sieve was passed and the filtrate was collected. Centrifuge at 2000 rpm/min for 5 min at room temperature, discard the supernatant, resuspend in PBS, centrifuge, and the precipitate is the cells to be detected.

**Detection of the percentage of immune cells in the spleen.** After grinding and crushing the spleen with a syringe on a 100 μm cell sieve, the RMPI-1640 whole medium was used to collect the filtrate. The filtrate was subsequently collected, and the supernatant was discarded before the precipitate was collected through centrifugation. Subsequently, the precipitate was collected after undergoing two red-breaking cycles with erythrocyte lysate, followed by washing to remove tissue fibers and prepare the cells for testing.

**Table S1.** Primers used for qPCR.

**Table S2.** Relative LA content in WT and KD LLC cell and in supernatant.

**Table S3.** Relative fluorescence intensity of HIF-1α and ARG1 protein in RAW 264.7 cells.

**Table S4.** Relative ratio of M1/M2 phenotype.

**Table S5.** Relative content of cytokines in supernatant of WT and KD LLC cells.

**Table S6.** In vivo pharmacokinetic parameters of PDIC-AC in blood.

**Figure S1.** Synthesis process of PDIC-AC and PDIC-NAC.

**Figure S2.** The ^1^H NMR spectrum of PDIC-AC.

**Figure S3.** The ^13^C NMR spectrum of PDIC-AC.

**Figure S4.** High resolution ESI-MS of PDIC-AC.

**Figure S5.** The ^1^H NMR spectrum of PDIC-NAC.

**Figure S6.** The ^13^C NMR spectrum of PDIC-NAC.

**Figure S7.** High-resolution ESI-MS of PDIC-NAC.

**Figure S8.** Optimized electrostatic potential of PDIC-NAC (left) and PDIC-AC^+^ (right) by DFT calculation based on the B3LYP/6-31G (d, p) with unified threshold.

**Figure S9.** (A) The pKa of PDIC-AC. (B)The UV-Vis Spectra of PDIC-AC (40 μM) and (C) The size distribution of PDIC-AC (40 μM) in PBS (pH 7.4) +10%FBS solutions.

**Figure S10.** Statistics on PDHK1 gene expression in LUAD (lung adenocarcinoma) and LUSC (lung squamous cell carcinoma).

**Figure S11.** (A) Cell viability of LLC incubated with DCA. Cell viability of MLE-12 incubated with (B) DCA, (C) PDIC-AC, and (D) PDIC-NAC for 24 h. Data are shown as mean ± SD (*n* = 3).

**Figure S12.** Surface electrostatic potential distribution of PDHK1 Kinase (PDB code: 2Q8G).

**Figure S13.** (A-C) Molecular docking results of and PDIC-AC^+^ with PDHK2, PDHK3, PDHK4 kinase. (D) CETSA results of PDHK2, PDHK3, PDHK4 kinases treated by PDIC-AC in LLC cells. (E) Expression levels of *p*-PDHA1, (F) intracellular LA content in WT and KD-PDHK1 LLC cells after treatment of DCA, PDIC-AC and PDIC-NAC. (G-I) The thermal stability of the PDHK2-4 isoforms. Data represent mean ± SD (n = 3). Statistical comparisons were performed using one-way ANOVA. ***P* < 0.01; ****P* < 0.001.

**Figure S14.** Analysis of the pcDNA3.1-C-3×Flag Vector Map.

**Figure S15.** The partial sequence alignment of wild type PDHK1 (above) and mutant PDHK1 (bottom).

**Figure S16.** The quantification results of the (A) Flag tagged mutant PDHK1 and (B) Flag tagged wild type PDHK1 thermal stability in CETSA.

**Figure S17.** Results of the median effect concentration (EC_50_ value) of (A) DCA, (B) PDIC-AC, and (C) PDIC-NAC on PDHK1 enzyme activity. Data are shown as mean ± SD (*n* = 3).

**Figure S18.** (A) Western blot validation of PDHK1 expression levels in LLC cells after transfection for 48 h. (B) qPCR analysis validating the knockdown efficacy of PDHK1 at the mRNA level after transfection for 60 h. Data represent mean ± SD (n = 3). Statistical comparisons were performed using one-way ANOVA. ****P* < 0.001.

**Figure S19.** Immunofluorescence detection of (A) HIF-1α in RAW 264.7 after treatment by conditioned medium collected from LLC cells after treated with DCA and PDIC-NAC and (B&C) statistical results. Data represent mean ± SD (n = 3). Statistical comparisons were performed using one-way ANOVA. ns, not significant (*P* > 0.05).

**Figure S20.** Immunofluorescence detection of (A) ARG1 in RAW 264.7 after treatment by conditioned medium collected from LLC cells after treated with DCA and PDIC-NAC and (B&C) statistical results. Data represent mean ± SD (n = 3). Statistical comparisons were performed using one-way ANOVA. ns, not significant (*P* > 0.05); ***P* < 0.01.

**Figure S21.** (A) Flow cytometry detection of M1 or M2 macrophage phenotyping of RAW 264.7 after DCA, DCA+LA, PDIC-NAC, PDIC-NAC+LA treatment and (B&C) the quantification results. Data represent mean ± SD (n = 3). Statistical comparisons were performed using one-way ANOVA. ns, not significant (*P* > 0.05).

**Figure S22.** Detection of cytokines in conditioned medium after cultured RAW 264.7 cells collected from PDIC-AC treated KD-LLC cells. Data are shown as mean ± SD (*n* = 3).

**Figure S23.** Level of LA in the supernatant of LLC cells following treatment with DCA, PDIC-AC and PDIC-NAC subsequent LA supplementation. Data represent mean ± SD (n = 3). Statistical comparisons were performed using one-way ANOVA. ns, not significant (*P* > 0.05).

**Figure S24.** (A) Immunofluorescence detection and (B) quantitative results of HIF-1α protein expression in RAW 264.7, which was incubulated by supernatant of KD LLC cells that treated with DCA (2 mM), PDIC-AC (2 μM) or PDIC-NAC (2 μM) for 12 h. Data represent mean ± SD (n = 3). Statistical comparisons were performed using one-way ANOVA. **P* < 0.05.

**Figure S25.** (A) Immunofluorescence detection and (B) quantitative results of ARG1 protein expression in RAW 264.7, which was in-cubulated supernatant of KD LLC cells that treated with DCA (2 mM), PDIC-AC (2 μM) or PDIC-NAC (2 μM) for 12 h. Data represent mean ± SD (n = 3). Statistical comparisons were performed using one-way ANOVA. ns, not significant (*P* > 0.05).

**Figure S26.** WB detection of intracellular HIF-1α and ARG1 expression level in RAW 264.7 cells after incubated with conditioned medium collected from KD LLC cells.

**Figure S27.** (A) Macrophage typing detection by flow cytometry and after the incubation with DCA, PDIC-AC and PDIC-NAC on KD PDHK1 LLC cells (B) quantitative results. Data represent mean ± SD (n = 3). Statistical comparisons were performed using one-way ANOVA. ns, not significant (*P* > 0.05).

**Figure S28.** The levels of IL-6, IL-12, TNF-α, and IL-10 secreted by the macrophages after incubating with the supernatants of WT-LLC cells. Data represent mean ± SD (n = 3). Statistical comparisons were performed using one-way ANOVA. ns, not significant (*P* > 0.05); **P* < 0.05; ***P* < 0.01.

**Figure S29.** The fluorescence quantification of total ROS in LLC cells after incubation of DCA (2 mM), PDIC-AC (2 μM) and PDIC-NAC (2 μM) for 8 h. Data represent mean ± SD (n = 3). Statistical comparisons were performed using one-way ANOVA. ****P* < 0.001.

**Figure S30.** (A) Flow cytometry analysis and (B) fluorescence quantification of total ROS in LLC cells after incubation of DCA (2 mM), PDIC-AC (2 μM) and PDIC-NAC (2 μM) for 8 h. Data represent mean ± SD (n = 3). Statistical comparisons were performed using one-way ANOVA. ****P* < 0.001.

**Figure S31.** Representative fluorescence images and quantification of (A) H_2_O_2_, (B) O_2_^•-^, (C) ^•^OH in LLC cells after incubation of DCA (2 mM), PDIC-AC (2 μM) or PDIC-NAC (2 μM) for 8 h. Data represent mean ± SD (n = 3). Statistical comparisons were performed using one-way ANOVA. ****P* < 0.01.

**Figure S32.** Flow cytometry analysis and quantification of (A) O_2_^•-^, (B) H_2_O_2_, (C) ^•^OH in LLC cells after incubation of DCA (2 mM), PDIC-AC (2 μM) or PDIC-NAC (2 μM) for 8 h. Data represent mean ± SD (n = 3). Statistical comparisons were performed using one-way ANOVA. ****P* < 0.001.

**Figure S33.** Relative expression of (A) D-LOOP, (B) Cyt b, (C) ND4, (D) Cox III and (E) ATP synthase subunits in PBS, DCA, PDIC-AC and PDIC-NAC treatment groups was determined by qPCR and normalized to ACTB. Data represent mean ± SD (n = 3). Statistical comparisons were performed using one-way ANOVA. **P* < 0.05; ***P* < 0.01; ****P* < 0.001.

**Figure S34.** Quantitative analysis of JC-1 aggregates/JC-1 monomers of LLC cells. Data represent mean ± SD (n = 3). Statistical comparisons were performed using one-way ANOVA. ***P* < 0.01; ****P* < 0.001.

**Figure S35.** (A) Basal Respiration, (B) Maximal Respiration, (C) ATP Production, and (D) Spare respiratory capacity after different treatments. Data represent mean ± SD (n = 3). Statistical comparisons were performed using one-way ANOVA. **P* < 0.05; ***P* < 0.01; ****P* < 0.001.

**Figure S36.** Quantitative results of LLC cell examined by flow cytometry. Data are shown as mean ± SD (*n* = 3).

**Figure S37.** (A) Flow cytometric analysis of apoptosis ratios in LLC cells following treatment with PDIC‑AC combined with exogenous ATP or methyl pyruvate and (B) corresponding quantitative results. Data represent mean ± SD (n = 3).

**Figure S38.** Western blot detection of apoptosis-related proteins.

**Figure S39.** (A) Representative immunofluorescence images of the externalization of CRT (red) and (B) HMGB1 release (red) in LLC cells. (C) The quantification of HMGB1 fluorescence intensity upon treatment by PDIC-AC at different concentrations. (D) The intracellular ATP content in LLC cells. Data represent mean ± SD (n = 3). Statistical comparisons were performed using one-way ANOVA. ns, not significant (*P* > 0.05); **P* < 0.05; ***P* < 0.01; ****P* < 0.001.

**Figure S40.** (A) Flow cytometry detection of the DC cells and (B) quantitative results. Data represent mean ± SD (n = 3). Statistical comparisons were performed using one-way ANOVA. ns, not significant (*P* > 0.05); ****P* < 0.001.

**Figure S41.** Flow cytometry detection of the helper T cells, CD8^+^ T cells and activated CD8^+^ T cells in (A) spleen and (B) in tumors.

**Figure S42.** Biosafety evaluation of PDIC-AC and PDIC-NAC. (A) The survival rate, (B) body weight, and (C) blood routine and blood biochemical index of the mice treated with PDIC-AC or PDIC-NAC at the dose of 2, 5, and 10 mg kg^-1^. WBC (White blood cell count), RDW (Red blood cell distribution width), RBC (Red blood cell count), AST (Aspartate aminotransferase), Mon% (Proportion of monocyte), MCV (Mean corpuscular volume), HCT (Hematocrit), Gran (Neutrophil granulocyte), CK (Creatine kinase), ALT (Alanine aminotransferase), MCH (mean corpuscular hemoglobin), MPV (Mean platelet volume). Data are shown as mean ± SD (*n* = 3).

**Figure S43.** H&E staining images of main organs from mice treated with PDIC-AC (2, 5, 10 mg/kg).

**Figure S44.** Fluorescence images of main organs in vitro collected from mice after intravenously injected PDIC-AC at the dose of 2 mg kg^-1^ body weight.

**Figure S45.** Body weight changes of mice in each group during the treatment period. Data represent mean ± SD (n = 5).

**Figure S46.** Quantitative results of macrophage phenotyping in lung tissue. Data represent mean ± SD (n = 3). Statistical comparisons were performed using one-way ANOVA. ***P* < 0.01; ****P* < 0.001.

**Figure S47.** Flow cytometry analysis and quantification of the proportion of (A) CD3^+^ CD4^+^ T cells, (B) CD3^+^ CD8^+^ T cells, and (C) activated CD8^+^ T cells in mice bearing primary lung tumors. Data represent mean ± SD (n = 3). Statistical comparisons were performed using one-way ANOVA. **P* < 0.05; ***P* < 0.01; ****P* < 0.001.

**Figure S48.** Flow cytometry analysis and quantification of the proportion of (A) CD3^+^ CD4^+^ T cells, (B) CD3^+^ CD8^+^ T cells, and (C) activated CD8^+^ T cells in spleens from a distant tumor model. Data represent mean ± SD (n = 3). Statistical comparisons were performed using one-way ANOVA. ***P* < 0.01; ****P* < 0.001.

**Figure S49.** (A) Flow cytometry detection of macrophage phenotyping in distant tumors and (B) quantitative results. Data represent mean ± SD (n = 3). Statistical comparisons were performed using one-way ANOVA. **P* < 0.01; ***P* < 0.01; ****P* < 0.001.

**Figure S50.** H&E images of the heart, liver, spleen, and kidneys of different treatment groups in the distant tumor.

**Figure S51.** Blood routine and blood biochemical index of the mice treated with different treatments in the LLC hormonal model. HGB (Hemoglobin), RDW (Red blood cell distribution width), RBC (Red blood cell count), PDW (Platelet distribution width), MCV (Mean corpuscular volume), MCH (Mean corpusular hemoglobin), HCT (Hematocrit), WBC (White blood cell count), Gran (Neutrophil granulocyte), Mon% (Percentage of Monocyte), Lymph% (Percentage of Lymphocyte), Lymph (Lymphocyte), AST (Aspartate aminotransferase). ALT (Alanine aminotransferase). Data are shown as mean ± SD (*n* = 3).
